# Characterization of ribosome stalling and no-go mRNA decay stimulated by the fragile X protein, FMRP

**DOI:** 10.1016/j.jbc.2024.107540

**Published:** 2024-07-04

**Authors:** MaKenzie R. Scarpitti, Benjamin Pastore, Wen Tang, Michael G. Kearse

**Affiliations:** Department of Biological Chemistry and Pharmacology, Center for RNA Biology, The Ohio State University, Columbus, Ohio, USA

**Keywords:** neurological disease, ribosome, RNA binding protein, translation control, translation regulation

## Abstract

Loss of functional fragile X mental retardation protein (FMRP) causes fragile X syndrome and is the leading monogenic cause of autism spectrum disorders and intellectual disability. FMRP is most notably a translational repressor and is thought to inhibit translation elongation by stalling ribosomes as FMRP-bound polyribosomes from brain tissue are resistant to puromycin and nuclease treatment. Here, we present data showing that the C-terminal noncanonical RNA-binding domain of FMRP is essential and sufficient to induce puromycin-resistant mRNA•ribosome complexes. Given that stalled ribosomes can stimulate ribosome collisions and no-go mRNA decay (NGD), we tested the ability of FMRP to drive NGD of its target transcripts in neuroblastoma cells. Indeed, FMRP and ribosomal proteins, but not poly(A)-binding protein, were enriched in isolated nuclease-resistant disomes compared to controls. Using siRNA knockdown and RNA-seq, we identified 16 putative FMRP-mediated NGD substrates, many of which encode proteins involved in neuronal development and function. Increased mRNA stability of four putative substrates was also observed when either FMRP was depleted or NGD was prevented *via* RNAi. Taken together, these data support that FMRP stalls ribosomes but only stimulates NGD of a small select set of transcripts, revealing a minor role of FMRP that would be misregulated in fragile X syndrome.

Loss of functional fragile X mental retardation protein (FMRP) causes fragile X syndrome (FXS) (1, 2, 3, 4) and is the leading monogenic cause of autism spectrum disorders and intellectual disability (5). FMRP is an RNA-binding protein that is highly expressed in the brain and gonads of both males and females (6); however, males are more phenotypically affected due to the X-linked mutation. While the complete molecular mechanism of FMRP has been disputed, most data support that FMRP acts as a translational repressor by inhibiting elongating ribosomes (7, 8, 9, 10, 11), yet FMRP may also inhibit the initiation of specific mRNAs or in certain contexts (12, 13, 14). Thus, at least one facet of the FXS phenotype is believed to be caused by aberrant and de-repressed protein synthesis at neuronal synapses (15, 16, 17).

The ability of FMRP to act as an RNA-binding protein is centered in all proposed models of FMRP function (6). To bind RNA, FMRP harbors at least three RNA-binding elements, including two canonical and structured KH1 and KH2 domains, as well as a short RGG box motif within the long flexible C-terminal domain (CTD) (18, 19, 20). A third KH domain (*i.e.*, KH0) has been reported based off of structural homology (20) but does not have detectable RNA-binding ability *in vitro* (21). We and others have previously biochemically dissected human FMRP and reported that the RGG box motif and the adjacent CTD together are essential and sufficient to inhibit translation of multiple reporter mRNAs in rabbit reticulocyte lysate (RRL) (8, 9). Importantly, our recent work has shown that the RGG box motif and CTD together, but not separately, form a noncanonical RNA-binding domain (ncRBD) that allows broader RNA-binding ability than previously reported (9). This observation helps explain the inability to identify a consensus motif for FMRP target transcripts in cells.

Consistent with the role of FMRP inhibiting translation elongation in neurons, FMRP is in association with puromycin- and RNase-resistant polyribosome complexes isolated from mouse brain (7). Since the ability of puromycin to act as a polypeptide chain terminator is specific for actively elongating ribosomes or those with an unoccupied A site (22), the puromycin-resistant complexes are thought to be translationally stalled. This is supported by the tight ribosome packing that is resistant to nuclease treatment. In alignment with this model, our recent work has shown that human FMRP inhibits the translation of nanoLuciferase (nLuc) reporter mRNA and causes the accumulation of ribosomes on reporter mRNA *in vitro*. Importantly, our *in vitro* FMRP-mediated translational control system faithfully recapitulates the formation of puromycin-resistant ribosomes on reporter mRNAs with human FMRP (9, 23) similar to what has been reported in brain tissue (7).

It is now widely understood that stalled ribosomes often trigger ribosome collisions that subsequently stimulate the no-go mRNA decay (NGD) pathway. In such examples, the leading ribosome is inactive and sterically hinders trailing ribosomes (24, 25). The interface between collided ribosomes forms a unique substrate for the ubiquitin E3 ligase ZNF598 (26) (Hel2 in yeast (27, 28)). After ubiquitylation of small ribosomal subunit proteins RPS10 and RPS20 by ZNF598 (29), an endonuclease (Cue2 in yeast (30), NONU-1 in *Caenorhabditis elegans* (31), and likely N4BP2 in humans (30)) is recruited to cleave the mRNA between collided ribosomes. Ribosome collisions and NGD are generally thought to be a quality control mechanism for cells to identify and destroy damaged or truncated mRNAs that, if translated, may be toxic. However, given that multiple lines of evidence support the ability of FMRP to stall elongating ribosomes, it is plausible, yet untested, that one role of FMRP is to induce ribosome collisions and NGD to negatively regulate gene expression of its target mRNAs.

In this report, we test and characterize the ability of FMRP to form puromycin-resistant mRNA•ribosome complexes and to cause ribosome collisions *in vitro* in RRL, as well as to stimulate NGD in neuroblastoma cells. Using a series of biochemical approaches, RNAi, and RNA-seq, the data presented here support that FMRP does stall ribosomes and stimulates NGD in neuroblastoma cells on a small number of transcripts, revealing a minor role of FMRP that would be misregulated in FXS when FMRP is lost.

## Results

### The ncRBD of FMRP, composed of the RGG box motif and the CTD, is essential and sufficient to stall ribosomes *in vitro*

**Figure 1:**
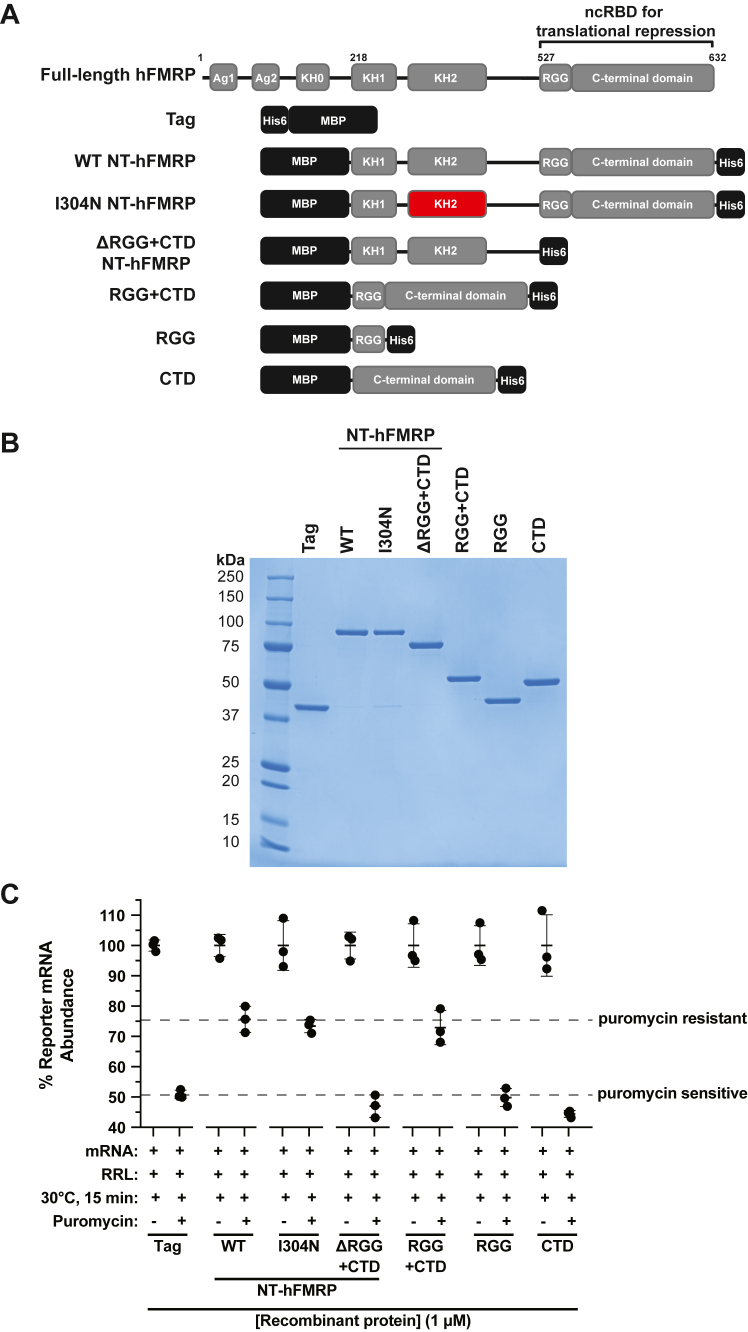
**The noncanonical RBD is essential and sufficient to cause puromycin-resistant mRNA•ribosome complexes *in vitro*.***A*, schematic of full-length human FMRP and recombinant N-terminally truncated human FMRP. Mutated/truncated domains are highlighted in *red*. The noncanonical RBD (ncRBD) required and sufficient for translational repression is shown on consists of residues 527–632 of full-length human FMRP (9). *B*, coomassie stain of recombinant proteins. *C*, relative quantification of nLuc reporter mRNA pelleted through a 35% (w/v) sucrose cushion after a low-speed centrifugation. nLuc•recombinant protein mRNPs were translated in RRL and treated with 0.1 mM puromycin (final) before being overlayed on the cushion and low-speed centrifugation. Final concentration of recombinant protein was 1 μM. Data are shown as mean ± SD. n = 3 biological replicates. Comparisons of the puromycin-treated samples were made using a two-tailed unpaired *t* test with Welch’s correction. Exact *p*-values are listed in Table S1.

Recombinant N-terminally truncated human FMRP (NT-hFMRP) (Fig. 1, *A* and *B*) has been shown to be more stable than recombinant full-length FMRP and retains translational repression activity (32). Using NT-hFMRP, we previously reported that FMRP inhibits translation, causes the accumulation of ribosomes on translationally repressed transcripts, and forms puromycin-resistant mRNA•ribosome complexes *in vitro* (9). Additionally, we have detailed that the RGG box motif and the CTD together form an ncRBD which elicits broad RNA-binding ability. Here, we use our published puromycin-induced mRNA•ribosome dissociation assay (9, 23) with nLuc reporter mRNPs formed with WT and mutant FMRP to test and confirm that the translationally repressive ncRBD of FMRP also drives the formation of puromycin-resistant mRNA•ribosome complexes (Figs. 1, *A* and *B* and S1). This assay takes advantage of the tyrosyl-tRNA mimic, puromycin, to act as a chain terminator of translation and to cause dissociation of the mRNA from ribosomes that are actively elongating or are in the unrotated/classic state with an unoccupied A site (22, 33, 34, 35). Reporter mRNA is carried into the ribosome pellet through a sucrose cushion if the ribosome is puromycin-resistant and stays bound to the mRNA. As expected, the control reaction with nLuc mRNA and control recombinant Tag protein, which does not inhibit translation (9), was sensitive to puromycin as indicated by the recovery of only ∼50% of reporter mRNA in the ribosome pellet (Fig. 1*C*). As previously shown (9), WT NT-hFMRP did cause puromycin-resistant mRNA•ribosome complexes (Fig. 1*C*). Notably, two rare FXS mutations, I304N (a missense mutation in the KH2 domain) and ΔRGG+CTD (a guanine insertion within the RGG box causing a frameshift and truncated isoform), that we previously shown to have different effects on translational repression (9), had opposing effects in the puromycin-induced mRNA•ribosome dissociation assay. I304N NT-hFMRP inhibits translation (9) and causes puromycin-resistant mRNA•ribosome complexes, similar to WT NT-hFMRP (Fig. 1). Conversely, ΔRGG+CTD NT-hFRMP does not inhibit translation (9) and causes puromycin-sensitive mRNA•ribosome complexes (Fig. 1).

We now confirm that the ncRBD is essential and sufficient to not only inhibit translation (9) but to also form puromycin-resistant mRNA•ribosome complexes (Fig. 1). The RGG box and the CTD alone formed puromycin-sensitive mRNA•ribosome complexes. However, the RGG box and CTD together, forming the ncRBD, resulted in puromycin-resistant mRNA•ribosome complexes to similar levels as seen for WT NT-hFMRP (Fig. 1).

### FMRP-mediated stalled ribosomes are distinct from mRNA structure–mediated stalled ribosomes

**Figure 2:**
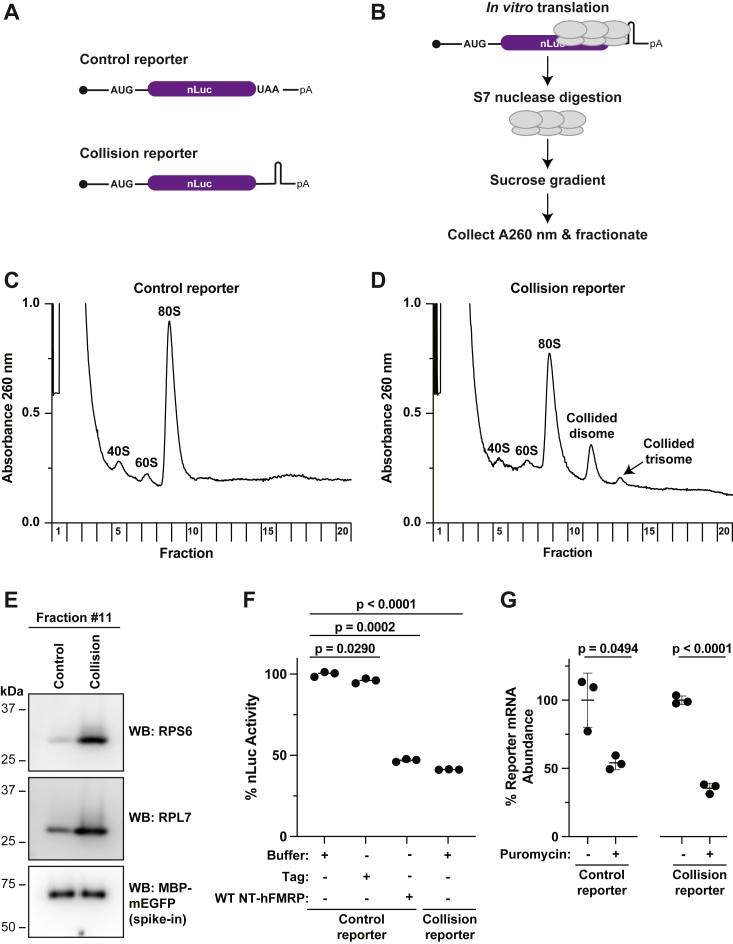
**Ribosomes stalled due to mRNA structure are puromycin-sensitive.***A*, schematic of control and collision reporter mRNAs. The control reporter harbors a stop codon, and the collision reporter lacks a stop codon but harbors a large GC-rich hairpin downstream of the nLuc coding sequence. *B*, schematic of workflow to detect S7/micrococcal nuclease-resistant ribosome collisions on reporter mRNAs in RRL. *C* and *D*, polysome analysis of translated control (*C*) and collision reporter mRNAs (*D*) in the absence of recombinant FMRP with nuclease treatment. The collision reporter mRNA generates nuclease-resistant disomes and trisomes, with a concurrent decrease in 80S monosomes as compared to the control reporter mRNA. *E*, anti-RPS6 and anti-RPL7 Western blots of fraction #11 (disome peak) which contains nuclease-resistant disomes. Recombinant MBP-mEGFP was spiked in and used as a loading control. *F*, *in vitro* translation of control and collision reporter mRNAs pre-incubated with protein storage buffer (Buffer) or the indicated recombinant protein (1 μM final). *G*, relative quantification of nLuc control and collision reporter mRNAs pelleted through a 35% (w/v) sucrose cushion after a low-speed centrifugation. mRNAs were translated in RRL in the absence of recombinant FMRP and treated with 0.1 mM puromycin (final) before being overlayed on the cushion and low-speed centrifugation. Data are shown as mean ± SD. n = 3 biological replicates. Comparisons were made using a two-tailed unpaired *t* test with Welch’s correction.

It is widely believed that stalled ribosomes often cause ribosome collisions, which in turn can stimulate NGD. One method used in the field to drive robust formation of stalled and collided ribosomes on reporter mRNAs is to delete the stop codon and insert a large GC-rich hairpin downstream (Fig. 2*A*). Collided ribosomes can then be detected by treating lysates with an endonuclease (*e.g.*, S7/micrococcal nuclease) and subsequent separation on sucrose gradients. The closely packed collided ribosomes occlude the nuclease from digesting the mRNA between ribosomes, enabling the detection of stable collided ribosomes in the disome or trisome fractions. Indeed, when compared to the control reporter mRNA, deletion of the stop codon and insertion of the hairpin in the collision reporter mRNA did result in nuclease-resistant disomes and trisomes in RRL (Figs. 2, *B*–*E* and S2). However, despite demonstrating that recombinant NT-hFMRP inhibits translation, causes the accumulation of ribosomes on translationally repressed transcripts, and forms puromycin-resistant mRNA•ribosome complexes in RRL (9) (Fig. 1), we were unable to detect nuclease-resistant collided ribosomes (*e.g.*, disomes) in inhibited translation reactions using both nLuc and Firefly Luciferase (FFLuc) reporter mRNAs across multiple concentrations of FMRP (Figs. S3–S6). RRL may lack brain-specific factors that stabilize or form FMRP-induced nuclease-resistant disomes. Additionally, FMRP may induce ribosome stalls closer to the 5ʹ end of the ORF which would limit disome formation or create collided ribosomes that are still sensitive to nuclease treatment.

Nevertheless, the collision reporter does allow us to assess whether differentially stalled ribosomes (*i.e.*, FMRP-mediated *versus* structure-mediated) are distinct. Consistent with its inability to stimulate termination and subsequent formation of collided ribosomes, the collision reporter mRNA was translated ∼3-fold less compared to the control reporter mRNA (both in the absence of FMRP) (Fig. 2*F*, lanes 1 *versus* 4). A similar decrease in translation was seen with WT-hFMRP–mediated inhibition on the control reporter mRNA (Fig. 2*F*, lanes 2 *versus* 3).

Using the same puromycin-induced mRNA•ribosome dissociation assay as described above, we next compared ribosomes inhibited by FMRP on the control reporter mRNA to ribosomes that were stalled by the hairpin on the collision reporter mRNA without FMRP. The mRNA•ribosome complexes formed by NT-hFMRP–mediated inhibition of the control reporter were resistant to puromycin (Fig. 1*C*). Conversely, the mRNA•ribosome complexes formed on the collision reporter mRNA in the absence of FMRP were sensitive to puromycin (Fig. 2*G*, right), which was similar to what was observed for mRNA•ribosome complexes formed on the control reporter mRNA in the absence of FMRP (Fig. 2*G*, left). It should be noted that *bona fide* inhibited and stalled ribosomes *via* emetine treatment are hypersensitive to puromycin (36, 37, 38). This is most likely due to the ribosome being in a state that more optimally accepts puromycin as a substrate. Together, these data suggest that FMRP-mediated stalled ribosomes are distinct from mRNA structure–mediated stalled ribosomes.

### FMRP regulates the stability of only a small set of targeted transcripts in cells

We next asked if endogenous FMRP cosediments with nuclease-resistant collided ribosomes in Neuro2A (N2A) cells, as was shown in brain lysates (7). S7 nuclease treatment did collapse polysomes into monosomes (Figs. S7, *A* and *B* and S8) and shifted signal for ribosomal proteins S6 and L7 from polysomes to monosomes on subsequent Western blots (Fig. S7, *C*–*F*). When probing the fraction that corresponds to collided disomes (*i.e.*, fraction #11), endogenous FMRP, RPS6, and RPL7 were significantly increased in the disome fraction in the nuclease-treated samples compared to the control (non-nuclease treated) samples (Fig. S7, *G*–*I*). This is consistent with FMRP inhibiting elongating ribosomes and causing them to collide. Importantly, this pattern was specific to ribosomal proteins and FMRP; poly(A)-binding protein (PABPC1) was depleted from the collided disome fraction upon nuclease treatment as expected since it should not be enriched on collided ribosomes (Fig. S7, *G*–*I*).

**Figure 3:**
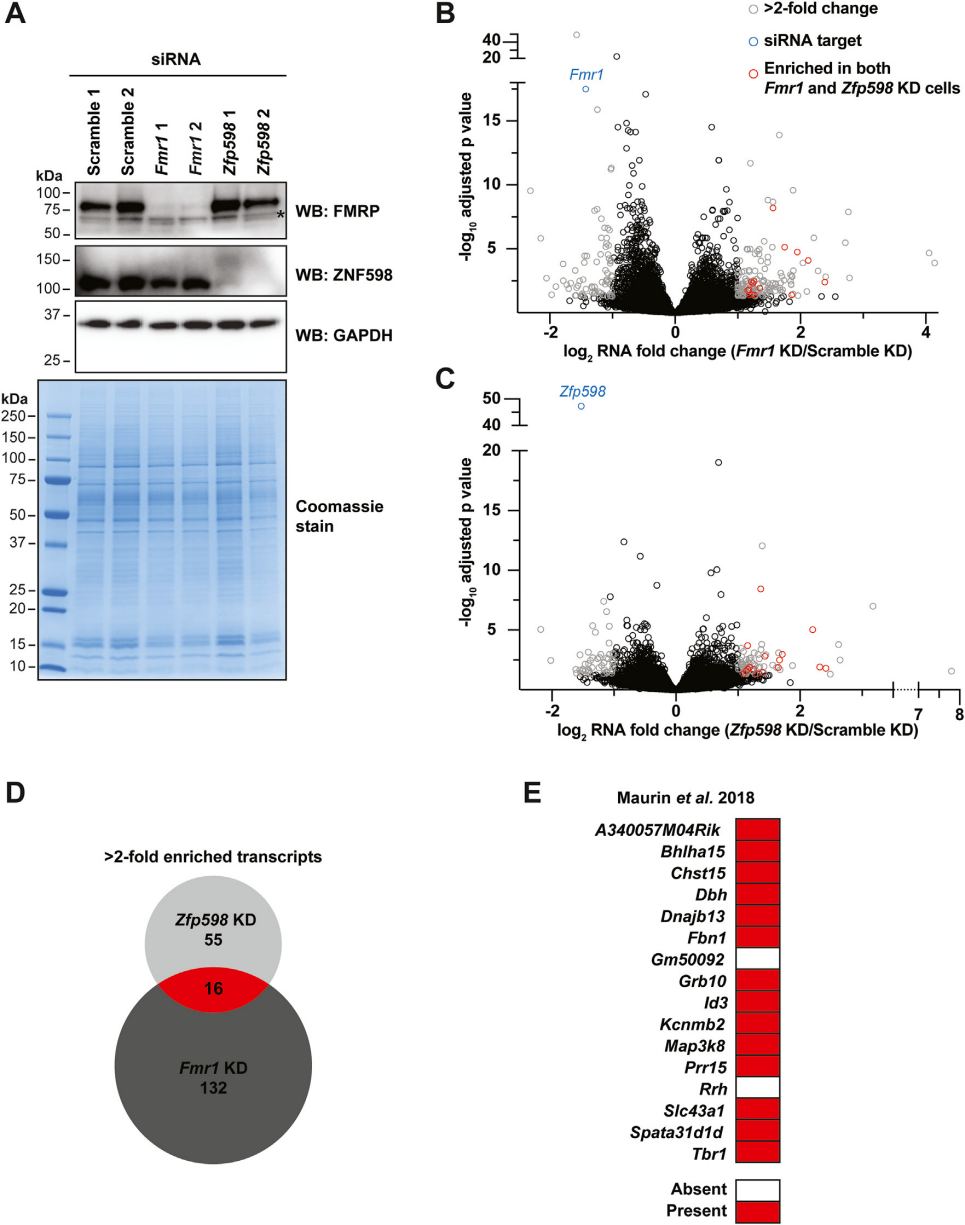
**RNA-seq reveals 16 transcripts as putative FMRP-mediated NGD targets in mouse N2A cells.***A*, anti-FMRP and anti-ZFP98 Western blots of N2A cell lysates treated with the indicated siRNAs. GAPDH was used as a loading control. ∗Denotes nonspecific immunoreactivity. Whole cell lysates were also separated by SDS-PAGE and stained with Coomassie as an additional loading control. *B* and *C*, volcano plots of RNA-seq data comparing Scramble control to *Fmr1* KD (*B*) or *Znf598* KD (*C*). Two independent siRNAs were used in triplicate per target for n = 6 per target. Transcripts enriched >2-fold (adjusted *p* < 0.05) in both *Fmr1* and *Zfp598* KD conditions are highlighted in *red*. *D*, venn diagram of >2-fold enriched transcripts in *Fmr1* and *Zfp598* KD conditions. Sixteen transcripts are enriched in both KD conditions tested (*red*). *E*, 14 of the 16 identified transcripts (*red* in *D*) are found in mouse brain FMRP CLIP-seq in Maurin *et al.* (39).

We next asked if FMRP could stimulate NGD of its target transcripts in N2A cells. We rationalized that if FMRP does have this function, depletion of FMRP should increase steady-state levels of its NGD-regulated transcripts. Depletion of the early and essential NGD factor, Zfp598 (ZNF598 in human cells, Hel2 in yeast), should cause an increase in all *bona fide* NGD targets. Thus, the overlapping transcripts that increase would be putative FMRP-mediated NGD substrates. To test this hypothesis, we performed RNA-seq of cells after 72 h knockdown (KD) with two independent Scramble control siRNAs, two independent siRNAs targeting *Fmr1*, or two independent siRNAs targeting *Zfp598* (Fig. 3*A*). To increase the statistical power of the analysis and gain higher confidence, we performed KD in triplicate per independent siRNA and then pooled the RNA-seq data for each KD condition (*i.e.*, Scramble KD, *Fmr1* KD, and *Zfp598* KD), for a combined n = 6 per condition (Fig. 3, *B* and *C*). As expected and in agreement with Western analysis (Fig. 3*A*), each target was significantly depleted in their respective KD samples (Fig. 3, *B* and *C*, blue data points). Using a two-fold RNA fold change cut off (adjusted *p* < 0.05), we identified 132 and 55 transcripts that increased with *Fmr1* and *Zfp598* depletion, respectively (Fig. 3, *B* and *C*, Tables S2 and S3). Of these transcripts, 16 increased at least 2-fold in both KD conditions compared to the Scramble controls (Fig. 3, *B* and *C* red data points and Fig. 3*D*), representing putative FMRP-mediated NGD substrates. Upon mining published data sets, we found that 14 of the 16 putative FMRP-mediated NGD substrates have been identified in a mouse brain FMRP crosslinking immunoprecipitation (CLIP)-seq data set (Fig. 3*E* and Table S4) (39), suggesting that the observed effects are direct rather than secondary or downstream. To validate these results, we repeated the *Fmr1* and *Zfp598* knockdowns, individually and in combination, and measured steady state mRNA levels by RT-qPCR. Only four putative targets identified by RNA-seq were reproducible by RT-qPCR for both independent siRNAs (Fig. S9), namely *Id3*, *Dbh*, *Map3k8*, and *Tbr1*. Double knockdown of *Fmr1* and *Zfp598* did not result in increased levels over either single knockdown, suggesting that FMRP and ZFP598 act within the same decay pathway for these four transcripts.

**Figure 4:**
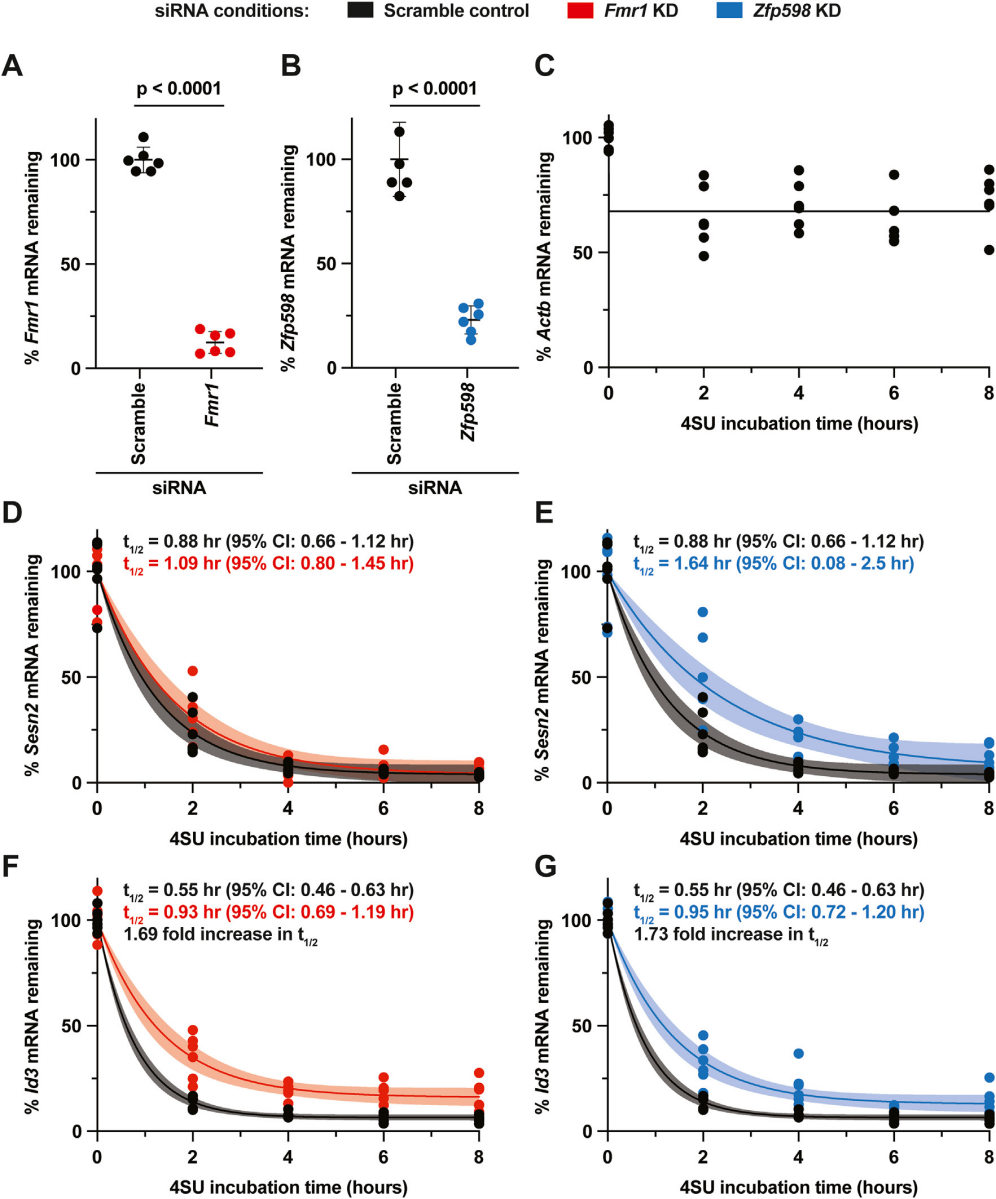
**Depletion of Fmr1 and the key no-go decay factor Zfp598 equally stabilize *Id3* mRNA in N2A cells.***A* and *B*, RT-qPCR was used to confirm depletion of *Fmr1* (*A*) and *Zfp598* (*B*) mRNA 72 h after KD at t = 0 h 4SU incorporation. Data are shown as the mean ± SD. n = 6 biological replicates. Comparisons were made using a two-tailed unpaired *t* test with Welch’s correction. *C*–*G*, roadblock-qPCR was used to measure mRNA half-lives (t_1/2_) of *Actb* mRNA (Scramble negative control) (*C*), as well as *Sesn2* mRNA (*D* and *E*) and *Id3* mRNA (*F* and *G*). The Scramble negative control is in *black* (and is the same in *D* and *E*, as well as in *F* and *G*), *Fmr1* KD is in *red*, and *Zfp598* KD is in *blue*. n = 6 biological replicates. mRNA t_1/2_ was determined by one phase decay trend lines calculated by nonlinear regression. The 95% confidence interval range is reported and is shown as a watermark.

To confirm the effects seen are due to changes in mRNA stability, we pursued measuring the change in mRNA half-life (t_1/2_) in N2A cells after *Fmr1* or *Zfp598* depletion using Roadblock-qPCR, which takes advantage of 4-thiouridine (4SU) incorporation into mRNA and the ability to form 4SU-*N*-ethylmaleimide adducts that block reverse transcriptase (40). Two independent siRNAs were used per KD target in triplicate, for a combined n = 6, per condition for each timepoint over an 8 h time course. Seventy-two hours after KD, N2A cells were treated with 4SU and total RNA was harvested at the indicated timepoints. In agreement with Western analysis and RNA-seq (Fig. 3, *A*–*C*), at t = 0 h, *Fmr1* and *Zfp598* mRNAs were significantly depleted in their respective KD samples (Fig. 4, *A* and *B*). Validating the Roadblock-qPCR approach in these cells, we did observe the robust stability of *Actb* mRNA (Fig. 4*C*) and instability of *Sesn2* mRNA (Fig. 4*D*, black data points and curve) over 8 h with the Scramble negative control siRNAs. Importantly, the short t_1/2_ of *Sesn2* mRNA was neither sensitive to *Fmr1* KD nor *Zfp598* KD when comparing the t_1/2_ 95% CI (Fig. 4, *D* and *E*). Importantly, we did observe a ∼1.7-fold increase that was statistically significant in the t_1/2_ of the FMRP-targeted *Id3* mRNA with both *Fmr1* and *Zfp598* KD. However, *Id3* mRNA was not completely stabilized over the 8 h time course, suggesting it is naturally unstable and/or under regulation of other mRNA decay pathways. Similar increased stability was observed for *Dbh*, *Map3k8*, and *Tbr1* mRNAs (Fig. S10), all of which were identified in our RNA-seq analysis as putative FMRP-mediated NGD substrates and were identified in a mouse brain FMRP CLIP-seq dataset (39). In total, these data provide evidence that FMRP can stall ribosomes to stimulate NGD on a small select set of targeted transcripts.

## Discussion

Data presented here add to the existing evidence that stalled ribosomes can have different properties depending on the effector. For example, emetine and cycloheximide bind to the E site of the 40S and 60S subunits of the 80S ribosome, respectively. Both inhibit and stall elongating ribosomes (41, 42), yet they have opposite effects on puromycylation (36, 37, 38, 42), where emetine enhances and cycloheximide inhibits puromycylation (36, 37, 38, 42). It is thought that emetine traps ribosomes in a state that more optimally accepts puromycin as a substrate (36, 37, 38). Here we show that ribosomes inhibited by FMRP and ribosomes inhibited by mRNA structure are distinct as only the former are puromycin-resistant.

High doses of translation elongation inhibitors stall most ribosomes preventing collisions, whereas intermediate doses cause a select few to act as roadblocks for trailing ribosomes (26, 43, 44, 45), ultimately causing ribosome collisions and NGD. Analogous to intermediate doses, in mining published datasets, we found that the 16 putative FMRP-mediated NGD substrates identified by RNA-seq represent low-density FMRP targets (39). High-density FMRP targets from the same and other FMRP CLIP-seq datasets (7, 39, 46, 47, 48, 49, 50, 51, 52), many of which are expressed in N2A cells, were not identified in our experiments as being regulated by NGD, suggesting that stimulating NGD is a minor function of FMRP. It is also possible that a high-density of bound FMRP inhibits most ribosomes and aids in preventing ribosome collisions. Furthermore, some data suggest that in neurons, most mRNAs are translated by monosomes (53), where ribosome collisions cannot be formed. Concordantly, neurons may have optimized their translation status to avoid causing widespread FMRP-mediated ribosome collisions and prevent mRNA instability in order to enable rapid regulated local translation in response to stimulation (54, 55).

The four putative FMRP-mediated NGD substrates that did show increased mRNA stability upon *Fmr1* and *Zfp598* knockdown do encode neuronal proteins (Table S4), all of which could contribute to documented FXS phenotypes (56), including those involved in neurotransmission (*Dbh*), cell differentiation regulation in the hippocampus (*Id3*), as well as neuronal migration and axonal projection (*Tbr1*). Notably, the Darnell group identified *Id3* as an FMRP target over 2 decades ago (57). ID3 is expressed in the hippocampus (58), an affected brain area in FXS (59), and binds to and inhibits the basic helix-loop-helix transcription factors, consequently inhibiting cell differentiation. Our data suggest that FMRP normally controls *Id3* mRNA stability. Thus, when FMRP is lost in FXS, the increased *Id3* mRNA levels could cause a concatenate increase in ID3 protein in the proliferative region of the hippocampus, resulting in aberrant inhibition of granule cell and dentate precursor cell differentiation (58).

## Experimental procedures

Full experimental procedures can be found in the Supporting Information. Exact *p*-values for Figure 1 can be found in Table S1. RNA-seq data analysis can be found in Tables S2 and S3. Reported gene functions for the hits in Figure 3 can be found in Table S4. Silencer Select siRNAs (Thermo Fisher Scientific) used in this study can be found in Table S5. Oligonucleotides used in this study can be found in Table S6.

## Data availability

All data is within the article or in the Supporting Information except for the raw RNA-seq data which has been deposited to the NCBI GEO under ascension number GSE254586.

## Supporting information

This article contains supporting information.

## Conflict of interest

The authors declare that they have no conflicts of interest with the contents of this article.
