# Supplementary material for: Characterization of ribosome stalling and no-go mRNA decay stimulated by the fragile X protein, FMRP
Source: J Biol Chem. 2024 Jul 4;300(8):107540. doi: 10.1016/j.jbc.2024.107540 (PMC11338112; doi:10.1016/j.jbc.2024.107540)
Supplement: Supporting Information [file mmc2.pdf]

SUPPORTING INFORMATION

Characterization of ribosome stalling and no-go mRNA decay stimulated by the Fragile X protein, FMRP

MaKenzie R. Scarpitti, Benjamin Pastore, Wen Tang, and Michael G. Kearse

SUPPLEMENTAL EXPERIMENTAL PROCEDURES

Plasmids

We have previously described the design of a G-quadruplex lacking nLuc coding sequence, that was based off of pNL1.1 (Promega), harboring the human β-globin 5' UTR that was synthesized by Integrated DNA Technologies and cloned into pcDNA3.1(+) (9). To generate a reporter that robustly stimulates ribosome collisions, the stop codon of nLuc was removed and a large GC-rich hairpin (HP) was inserted downstream using the Q5 Site-Directed Mutagenesis Kit (NEB # E0552S). pCRII/FFLuc, which contains the FFLuc coding sequence from pGL4.13 (Promega) downstream from the T7 RNA polymerase promoter, was previously described (Kearse *et al*, 2016).

An *E. coli* optimized coding sequence for human FMRP (isoform 1) was designed and synthesized by Genscript, and then subcloned into pET His6 MBP TEV LIC cloning vector (1M), which was a gift from Scott Gradia (Addgene plasmid # 29656), through ligation-independent cloning (LIC) using Novagen's LIC-qualified T4 DNA polymerase (Sigma # 70099-M) as described by Q3 Macrolab (http://qb3.berkeley.edu/macrolab/). The His6-tag was deleted from the N-terminus and inserted at the C-terminus. The NT-hFMRP sequence included a P451S mutation to prevent ribosome stalling at a poly-proline stretch and formation of truncated recombinant protein, as previously described (32). Point mutations and deletions were achieved using the Q5 Site-Directed Mutagenesis Kit. To be as consistent as possible across the previous literature, we refer to the RGG box motif as to the minimal region identified by Darnell and colleagues that bound Sc1 RNA with highest affinity (57).

All plasmids were propagated in TOP10 *E. coli* (Thermo Fisher # C404006), purified using the PureYield Plasmid Miniprep or Midiprep Systems (Promega # A1222 and A2495), and validated by Sanger sequencing at The Ohio State University Comprehensive Cancer Center Genomics Shared Resource (OSUCCC GSR). Sequences of the reporters and recombinant proteins are provided in the Supporting Information.

Reporter mRNA *in vitro* transcription

All nLuc plasmids were linearized with XbaI and purified using a Zymo DNA Clean & Concentrator 25 Kit (Zymo Research # D4065). pCRII/FFLuc was linearized with HindIII using a Zymo DNA Clean & Concentrator 25 Kit. DNA was transcribed into mRNA which was co-transcriptionally capped with the Anti-Reverse Cap Analog (ARCA) 3′-O-Me-m7G(5′)ppp(5′)G (NEB # S1411) using the HiScribe T7 High Yield RNA Synthesis Kit (NEB # E2040). Our standard 10 µL reactions used 0.5 µg of linear plasmid template and an 8:1 ARCA:GTP ratio. Reactions were incubated at 30°C for 2 hrs, then incubated with 20 U of DNaseI (NEB # M0303S) at 37°C for 15 min, and then purified using a Zymo RNA Clean & Concentrator 25 Kit (Zymo Research # R1018). Reporter mRNA was eluted in 75 µL of RNase-free water, aliquoted in single use volumes, and stored at -80°C. Reporter mRNA integrity was confirmed by denaturing formaldehyde agarose gel electrophoresis and ethidium bromide visualization. We routinely found the 30°C incubation resulted in less observable truncated products than incubation at 37°C and did not significantly affect yield for our purposes. mRNA translation in nuclease-treated rabbit reticulocyte lysate (RRL) is poly(A)-independent (Soto Rifo *et al*., 2007); thus, we omitted polyadenylating reporter mRNAs in this study.

Recombinant protein expression and purification

All recombinant proteins were expressed in Rosetta 2(DE3) *E. coli* (Sigma # 71397-4)

using MagicMedia *E. coli* Expression Medium (Thermo Fisher # K6803) supplemented with 50 µg/mL kanamycin and 35 µg/mL chloramphenicol for auto-induction. A 5 mL starter culture in LB media supplemented with 50 µg/mL kanamycin, 35 µg/mL chloramphenicol, and 1% glucose (w/v) was inoculated with a single colony and grown overnight at 37°C, 250 rpm. 1 mL of a warm and fresh overnight starter culture was then used to inoculate 50 mL of room temperature MagicMedia and incubated for 48-72 hrs at 18°C, 160 rpm in a 250 mL baffled flask. After auto-induction, cultures were pelleted and stored at -20°C for purification later. Recombinant proteins were purified using a dual affinity approach, first using the C-terminal His6-tag, then the N-terminal MBP-tag. Cell pellets were resuspended and lysed with BugBuster Master Mix (Sigma # 71456) using the recommended 5 mL per 1 g wet cell pellet ratio for 10 min at room temperature with gentle end-over-end rotation (10-15 rpm). Lysates were placed on ice and kept cold moving forward. Lysates were cleared by centrifugation for 20 min at 18,000 rcf in a chilled centrifuge (4°C). Lysates were then incubated with HisPur Cobalt Resin (Thermo Fisher # 89965) in a Peirce centrifugation column (Thermo # 89897) for 30 min at 4°C with gentle end-over-end rotation. Columns were centrifuged in a pre-chilled (4°C) Eppendorf 5810R for 2 min at 700 rcf to eliminate the flow through and then were washed 5X with two resin-bed volumes of ice-cold Cobalt IMAC Wash Buffer (50 mM Na_3PO_4, 300 mM NaCl, 10 mM imidazole; pH 7.4) in a pre-chilled (4°C) Eppendorf 5810R for 2 min at 700 rcf. His-tagged proteins were then eluted in a single elution step with two resin-bed volumes of ice-cold Cobalt IMAC Elution Buffer (50 mM Na_3PO_4, 300 mM NaCl, 150 mM imidazole; pH 7.4) by gravity flow. Eluates were then incubated with Amylose resin (NEB # E8021) in a centrifugation column for 2 hrs at 4°C with gentle end-over-end rotation (10-15 rpm). Columns were washed 5X with at least two bed-volumes of ice-cold MBP Wash Buffer (20 mM Tris-HCl, 200 mM NaCl, 1 mM EDTA; pH 7.4) by gravity flow. MBP-tagged proteins were then eluted by a single elution step with two resin-bed volumes of ice-cold MBP Elution Buffer (20 mM Tris-HCl, 200 mM NaCl, 1 mM EDTA, 10 mM maltose; pH 7.4) by gravity flow. Recombinant proteins were then desalted and buffer exchanged into Protein Storage Buffer (25 mM Tris-HCl, 125 mM KCl, 10% glycerol; pH 7.4) using a 7K MWCO Zeba Spin Desalting Column (Thermo Fisher # 89892) and, if needed, concentrated using 10K MWCO Amicon Ultra-4 (EMD Millipore # UFC803024). Recombinant protein concentration was determined by Pierce Detergent Compatible Bradford Assay Kit (Thermo Fisher # 23246) with BSA standards diluted in Protein Storage Buffer as well as SDS-PAGE and Coomassie staining before aliquoting in single use volumes, snap freezing in liquid nitrogen, and storage at -80°C.

mRNP formation and *in vitro* translation

In vitro translation was performed in the dynamic linear range as previously described but adapted to translate mRNPs (9, 23, and Kearse *et al*., 2016). 30 nM *in vitro* transcribed nLuc reporter mRNA was diluted in RNA Dilution Buffer (10 mM Tris-HCl, 5 mM $Mg(OAc)_2$, 100 mM KCl; pH 7.4). In a total of 4 µL, 30 fmol of nLuc reporter mRNA was mixed with 0-10 picomol of recombinant protein and 100 picomol of UltraPure BSA (Thermo Fisher # AM2618) on ice for 1 hr. UltraPure BSA stock was diluted in protein storage buffer and its addition was necessary to prevent non-specific binding of the reporter mRNA to the tube. For *in vitro* translation reactions, 6 µL of a Rabbit Reticulocyte Lysate (RRL) master mix was added to each 4µL mRNP complex. 10 µL *in vitro* translation reactions were performed in the linear range using 3 nM mRNA in the Flexi RRL System (Promega # L4540) with final concentrations of reagents at 30% RRL, 10 µM amino acid mix minus leucine, 10 µM amino acid mix minus Methionine, 0.5 mM $Mg(OAc)_2$, 100 mM KCl, 8 U murine RNase inhibitor (NEB # M0314), 0-1 µM recombinant protein, and 10 µM UltraPure BSA. Reactions were incubated for 30 min at 30°C, terminated by incubation on ice and diluted 1:5 in Glo Lysis Buffer (Promega # E2661). 25 µL of prepared Nano-Glo reagent (Promega # N1120) was mixed with 25 µL of diluted reaction and incubated at room

temperature for 5 min in the dark (with gentle shaking during the first minute), and then read on a Promega GloMax Discover Multimode Microplate Reader. FFLuc mRNA was treated and translated exactly the same; FFLuc luminescence was measured exactly the same but used ONE-Glo (Promega # E6110) instead of Nano-Glo.

mRNA•ribosome dissociation assays with puromycin

We have previously described in detail and in a complete methods manuscript the use of the ability of puromycin to dissociate ribosomes from reporter mRNA with a low-speed sucrose cushion (9, 23). nLuc reporter mRNA translation was performed as described above except that translation was limited to 15 min at 30°C. Samples were then placed on ice for 3 min before the addition of 0.1 mM puromycin (final) and further incubation at 30°C for 30 min. Control samples lacking puromycin (water added instead) were kept on ice. Cycloheximide (1.43 mg/mL final) was then added to all samples to preserve ribosome complexes on mRNAs and halt puromycin incorporation. In a separate tube, FFLuc reporter mRNA was translated as described above (3 nM mRNA conditions) for 15 min at 30°C and was terminated by the addition of 1.43 mg/mL cycloheximide (final) and incubation on ice.

The treated nLuc and FFLuc translation reactions (from above) were combined on ice and then mixed with an equal volume (28 µL) of ice-cold 2X Ribosome Dilution Buffer (40 mM Tris-HCl, 280 mM KCl, 20 mM $MgCl_2$, 200 µg/ml cycloheximide, 2 mM DTT; pH 7.4). The entire 56 µl volume was then layered on top of 130 µl of ice-cold 35% (w/v) buffered sucrose (20 mM Tris-HCl, 140 mM KCl, 10 mM $MgCl_2$, 100 µg/mL cycloheximide, 1 mM DTT; pH 7.4) in a pre-chilled 7 mm x 20 mm thick-walled polycarbonate ultracentrifuge tubes (Thermo Scientific # 45233) and centrifuged in a S100AT3 rotor at 4°C for 60 min at 50,000 x g (43,000 rpm) in a Sorvall Discovery M120 SE Micro-Ultracentrifuge. The supernatant was then discarded and each pellet was resuspended in 0.5 mL of TRIzol (Thermo Fisher # 15596018). Total RNA was extracted from each pellet following the manufacturer’s protocol with glycogen (Thermo Fisher # R0561) added at the isopropanol precipitation step. The resulting RNA pellet was resuspended in 30 µL nuclease-free water. 16 µL of extracted RNA was converted to cDNA using iScript Reverse Transcription Supermix for RT-qPCR (Bio-Rad # 1708841). cDNA reactions were then diluted 10-fold with nuclease-free water and stored at -20°C or used immediately. RT-qPCR was performed in 15 µL reactions using iTaq Universal SYBR Green Supermix (Bio-Rad # 1725124) in a Bio-Rad CFX Connect Real-Time PCR Detection System with 1.5 µL diluted cDNA and 250 nM (final concentration) primers. nLuc reporter mRNA abundance was normalized to the spiked-in control FFLuc mRNA using the Bio-Rad CFX Maestro software (ΔΔCt method). Abundance of total signal was calculated using $Q_n = 2^{\Delta\Delta Ct}$ and $P = 100 \times Q_n/Q_{total}$ as previously described (Pringle *et al.*, 2019). Primers for RT-qPCR can be found in **Table S6**.

Cell culture and siRNA knockdowns

N2A cells were obtained from ATCC (# CCL-131) and maintained in high glucose DMEM (Thermo # 11995065) supplemented with 10% heat-inactivated FBS and 1% penicillin-streptomycin in standard tissue culture-treated plastics at 37°C with 5% CO_2. N2A cells were seeded in 12-well plates 24 hrs prior to knockdown, and then transfected with Silencer Select siRNAs (Thermo) using Lipofectamine RNAiMAX (Thermo # 13778150) following the manufacturer’s recommendation. At the time of transfection, N2A cells were at ~20% confluency. 24 hrs post siRNA transfection, the media was changed. After 72 hr transfection, total RNA or protein was harvested by TRIzol or RIPA buffer, respectively. Silencer Select siRNAs used in this study are listed in **Table S5**.

Sucrose gradient ultracentrifugation

In vitro translation reactions were scaled up 10-fold to 100 µL. After 30 min at 30°C, reactions were transferred to ice, 1 µL of 100 mg/mL cycloheximide was added (~1 mg/mL final), and samples were snap frozen in liquid nitrogen and stored at -80°C. When ready to perform sucrose gradients, samples were then thawed on ice, 5 µL of 10 mM $CaCl_2$ (~0.5 mM final) and 5.3 µL of 3,000 U/mL S7 micrococcal nuclease (Thermo # EN0181; stock at 30,000 U/mL in PBS; ~150 U/mL final) was added. After nuclease digestion at 25°C for 10 min, samples were quenched by adding 2.2 µL of 50 mM EGTA (~1 mM final). Samples were then diluted with an equal volume of ice-cold 2X Polysome Dilution Buffer (40 mM Tris-HCl, 280 mM KCl, 20 mM $MgCl_2$, 2 mM DTT, 200 µg/mL cycloheximide; pH 7.4), gently mixed, and layered on top of a linear 10-50% (w/v) buffered sucrose gradient (20 mM Tris-HCl, 140 mM KCl, 10 mM $MgCl_2$, 1 mM DTT, 100 µg/mL cycloheximide; pH 7.4) in a 14 mm × 89 mm thin-wall Ultra-Clear tube (Beckman # 344059) that was formed using a Biocomp Gradient Master. Gradients were centrifuged at 35K rpm for 120 min at 4°C in a SW-41Ti rotor (Beckman) with maximum acceleration and no brake using a Beckman Optima L-90 Ultracentrifuge. Gradients were subsequently fractionated into 0.5 mL volumes using a Biocomp piston fractionator with a TRIAX flow cell (Biocomp) recording a continuous $A_{260\ nm}$ trace.

Per replicate, N2A cells were seeded in four 15 cm tissue culture treated plates and allowed to grow for 2-3 days until ~70% confluent. Cells were briefly treated with 100 µL/mL cycloheximide for 2 min, then rinsed with ice-cold PBS with 100 µL/mL cycloheximide, harvested using a cell lifter, pooled, pelleted at 500 rcf for 5 min at 4°C in a pre-chilled centrifuge, snap froze in liquid nitrogen, and stored at -80°C. Cell pellets were thawed on ice and then lysed in 950 µL ice-cold Polysome Lysis Buffer (20 mM Tris-HCl, 140 mM KCl, 10 mM $MgCl_2$, 1 mM DTT, 100 µg/mL cycloheximide, 1% (v/v) Triton X-100; pH 7.4) for 10 min on ice. Cell debris was then cleared at 13,000 rcf at 4°C in a pre-chilled microcentrifuge. The supernatant was taken and 450 µL was aliquoted into two new pre-chilled microcentrifuge tubes. 22.5 µL of 10 mM $CaCl_2$ (~0.5 mM final) and 2.4 µL of either 30,000 U/mL S7 micrococcal nuclease (~150 U/mL final) or PBS was added. After nuclease digestion at 25°C for 10 min, samples were quenched by adding 9.5 µL of 50 mM EGTA (~1 mM final). 400 µL was then layered on top of a linear 10-50% (w/v) buffered sucrose gradient (20 mM Tris-HCl, 140 mM KCl, 10 mM $MgCl_2$, 1 mM DTT, 100 µg/mL cycloheximide; pH 7.4), centrifuged, and fractionated as described above.

Western blotting

When assaying sucrose gradient fractions, 100 µL of the 500 µL collected fractions was added and mixed with 2 µL of 1.4 µM MBP-mEGFP, which served as a spike-in loading control. 34 µL of 4X reducing SDS sample buffer (Bio-Rad # 1610737) was then added, mixed, and heated at 70°C for 15 min. 30 µL was then separated by Tris-Glycine SDS-PAGE (Thermo # XP04200BOX) and transferred on to 0.2 µm PVDF membrane (Thermo # 88520). Membranes were then blocked with 5% (w/v) non-fat dry milk in TBST (1X Tris-buffered saline with 0.1% (v/v) Tween 20) for 30 min at room temperature before overnight incubation with primary antibodies in TBST at 4°C with gentle rotation/rocking. After three 10 min washes with TBST, membranes were incubated with HRP-conjugated secondary antibody in TBST for 1 hr at room temperature and then washed again with three 10 min washes with TBST. Chemiluminescence was performed with SuperSignal West Pico PLUS (Thermo # 34577) for GFP, RPS6, RPL7, and PABP and with SuperSignal West Atto Maximum Sensitivity Substrate (Thermo # A38555) for FMRP, and imaged using an Azure Sapphire Biomolecular Imager. Rabbit anti-GFP (Cell Signaling # 2956S) was used at 1:1,000. Rabbit anti-RPS6 (Cell Signaling # 2217) was used at

1:1,000. Rabbit anti-RPL7 (abcam # ab72550) was used at 1:1,000. Rabbit anti-PABP (abcam # ab21060) was used at 1:1,000. HRP-conjugated goat anti-rabbit IgG (H+L) (Thermo # 31460) was used at 1:10,000 for GFP and PABP, 1:100,000 for FMRP, and 1:30,000 for RPS6 and RPL7.

When assaying N2A cell lysates after 72 hr knockdown, whole cell lysates were prepared using RIPA buffer. Briefly, cells were placed on a bed of ice and media was aspirated before a gentle 1 mL ice-cold PBS rinse. Cells were then lysed in 300 µL of ice-cold RIPA buffer for 10 min at 4°C with gentle rocking. The entire lysate (including any apparent cell debris) was mixed with 100 µL of 4X reducing SDS sample buffer and heated to 70°C for 15 min. Samples were then homogenized by syringing 6X through a 28G needle. 30 µL was then separated by Tris-Glycine SDS-PAGE, transferred on 0.2 µm PVDF, and probed as described above. Rabbit anti-ZNF598 (Thermo # PA5-59777) was used at 1:1,000. Rabbit anti-FMRP (Abcam # ab17722) was used at 1:1,000. Rabbit anti-GAPDH (Cell Signaling # 5174) was used at 1:1,000. HRP-conjugated goat anti-rabbit IgG (H+L) (Thermo # 31460) was used at 1:10,000 for ZNF598 and FMRP, and at 1:30,000 for GAPDH. Chemiluminescence was performed with SuperSignal West Pico PLUS and imaged using an Azure Sapphire Biomolecular Imager.

RNA-seq and mRNA decay analyses via Roadblock-qPCR

After 72 hr knockdown, total RNA was extracted with TRIzol reagent following the manufacturer's protocol. RNA concentration and purity was determined by UV spectroscopy (i.e., Nanodrop). RNA library preparation (with RNA quality validation and rRNA depletion included) and sequencing was conducted by Novogene. Adapters and poorly sequenced reads were removed from raw sequencing data using TrimGalore (Kechin *et al*., 2017). The quality of sequencing reads following trimming and filtration was assessed using FastQC (Andrews, 2015). Processed sequencing reads were aligned to the mouse reference genome (GRCm39) using STAR (Dobin *et al*., 2013). Sequence coverage BigWig files were generated using STAR alignment BAM output using the deepTools tool bamCoverage, using counts per million (CPM) normalization (Ramirez *et al*., 2014). Genomic features were quantified by counting the number of reads aligning to exons of genes using FeatureCounts (Liao *et al*., 2013). Normalization and differential gene expression analysis was then preformed using the R package DESeq2 (Love *et al*., 2014). The pipeline used for the pre-processing, alignment, and post-alignment analysis of RNA-seq data can be found at https://doi.org/10.5281/zenodo.8302724. Any other custom scripts used in this analysis are available upon request. All raw RNA-seq data has been deposited in the NCBI Gene Expression Omnibus (GEO).

Roadblock-qPCR was used to determine mRNA half-lives and was performed as described by the Thoreen lab (40). 72 hr post knockdown, media was replaced with pre-warmed completed supplemented with 400 µM 4SU (stock at 80 mM in DMSO; Sigma #T4509-100MG). Timepoints were taken immediately (0 hr), 2, 4, 6, and 8 hrs later by aspirating media and adding 1 mL of TRIzol. After extracting total RNA by following the manufacture's recommendations, 3 µg of RNA was modified in NEM Reaction Buffer (50 mM Tris-HCl, 1 mM EDTA, and 50 mM NEM; pH8) in a 50 µL reaction at 42°C for 90 min. 1 M NEM (Sigma # 3876) was made with 100% EtOH, aliquoted, and stored at -20°C. The reaction was quenched by addition of 20 mM DTT (final) and RNA was purified using an RNA Clean & Concentrator-5 Kit (Zymo # R1013) with a final elution volume of 15 µL. 1 µg of recovered RNA was used to generate cDNA for RT-qPCR using the iScript Reverse Transcription Supermix for RT-qPCR (Bio-Rad # 1708841). cDNA reactions were then diluted 10-fold with nuclease-free water and stored at −20°C or used immediately. RT-qPCR was performed in 15 µl reactions using iTaq Universal SYBR Green Supermix (Bio-Rad# 1725124) in a Bio-Rad CFX Connect Real-Time

PCR Detection System with 1.5 µl diluted cDNA and 250 nM (final concentration) primers. Target mRNA levels were normalized to 18S rRNA, and half-lives were calculated using one phase decay trend lines calculated by nonlinear regression in GraphPad Prism 10.0.3. All data are reported and the 95% confidence interval was included as a watermark when appropriate.

Steady state mRNA levels in **Figure 4** and **Figure S9** were normalized to 18S rRNA and GAPDH, respectively.

All RT-qPCR primer sequences are available in **Table S6**.

SUPPLEMENTAL REFERENCES FOR EXPERIMENTAL PROCEDURES

Andrews, S. (2015) FastQC. https://qubeshub.org/resources/fastqc.

Dobin, A., Davis, C. A., Schlesinger, F., Drenkow, J., Zaleski, C., Jha, S. *et al*. (2013) STAR: ultrafast universal RNA-seq aligner. Bioinformatics **29**, 15-21 10.1093/bioinformatics/bts635

Kearse, M. G., Green, K. M., Krans, A., Rodriguez, C. M., Linsalata, A. E., Goldstrohm, A. C. *et al*. (2016) CGG Repeat-Associated Non-AUG Translation Utilizes a Cap-Dependent Scanning Mechanism of Initiation to Produce Toxic Proteins. Mol Cell **62**, 314-322 10.1016/j.molcel.2016.02.034

Kechin, A., Boyarskikh, U., Kel, A., and Filipenko, M. (2017) cutPrimers: A New Tool for Accurate Cutting of Primers from Reads of Targeted Next Generation Sequencing J Comput Biol **24**, 1138-1143 10.1089/cmb.2017.0096

Liao, Y., Smyth, G. K., and Shi, W. (2013) featureCounts: an efficient general purpose program for assigning sequence reads to genomic features. Bioinformatics **30**, 923-930 10.1093/bioinformatics/btt656

Love, M. I., Huber, W., and Anders, S. (2014) Moderated estimation of fold change and dispersion for RNA-seq data with DESeq2. Genome Biology **15**, 550 10.1186/s13059-014-0550-8

Pringle, E. S., McCormick, C., and Cheng, Z. (2019) Polysome Profiling Analysis of mRNA and Associated Proteins Engaged in Translation. Curr Protoc Mol Biol **125**, e79 10.1002/cpmb.79

Ramírez, F., Dündar, F., Diehl, S., Grüning, B. A., and Manke, T. (2014) deepTools: a flexible platform for exploring deep-sequencing data. Nucleic Acids Res **42**, W187-191 10.1093/nar/gku365

Soto Rifo, R., Ricci, E. P., Décimo, D., Moncorgé, O., and Ohlmann, T. (2007) Back to basics: the untreated rabbit reticulocyte lysate as a competitive system to recapitulate cap/poly(A) synergy and the selective advantage of IRES-driven translation. Nucleic Acids Res **35**, e121 10.1093/nar/gkm682

SUPPLEMENTAL FIGURES

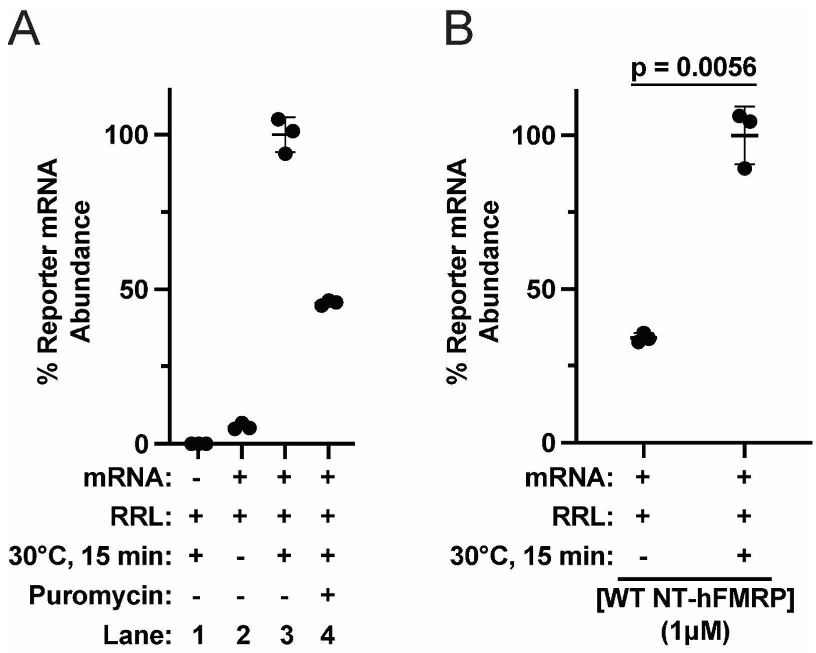

Figure S1. nLuc mRNA pelleting through the sucrose cushion is translation dependent. A) Relative quantification of nLuc reporter mRNA pelleted through a 35% (w/v) sucrose cushion after a low-speed centrifugation. Lane 1 is a negative control lacking nLuc mRNA. Lane 2 is a negative control containing mRNA in RRL but not incubated at 30°C to start translation. Lane 3 is nLuc mRNA in RRL translated for 15 min at 30°C. Lane 4 is nLuc mRNA in RRL translated for 15 min at 30°C and then incubated with 0.1 mM puromycin (final) for 30 min at 30°C. Data are shown as mean ± SD. n=3 biological replicates. B) Relative quantification of nLuc reporter mRNA pelleted through a 35% (w/v) sucrose cushion after a low-speed centrifugation with WT NT-hFMRP (1 μM final) with and without translation (15 min at 30°C). Data are shown as mean ± SD. n=3 biological replicates. Comparisons were made using a two-tailed unpaired t test with Welch's correction.

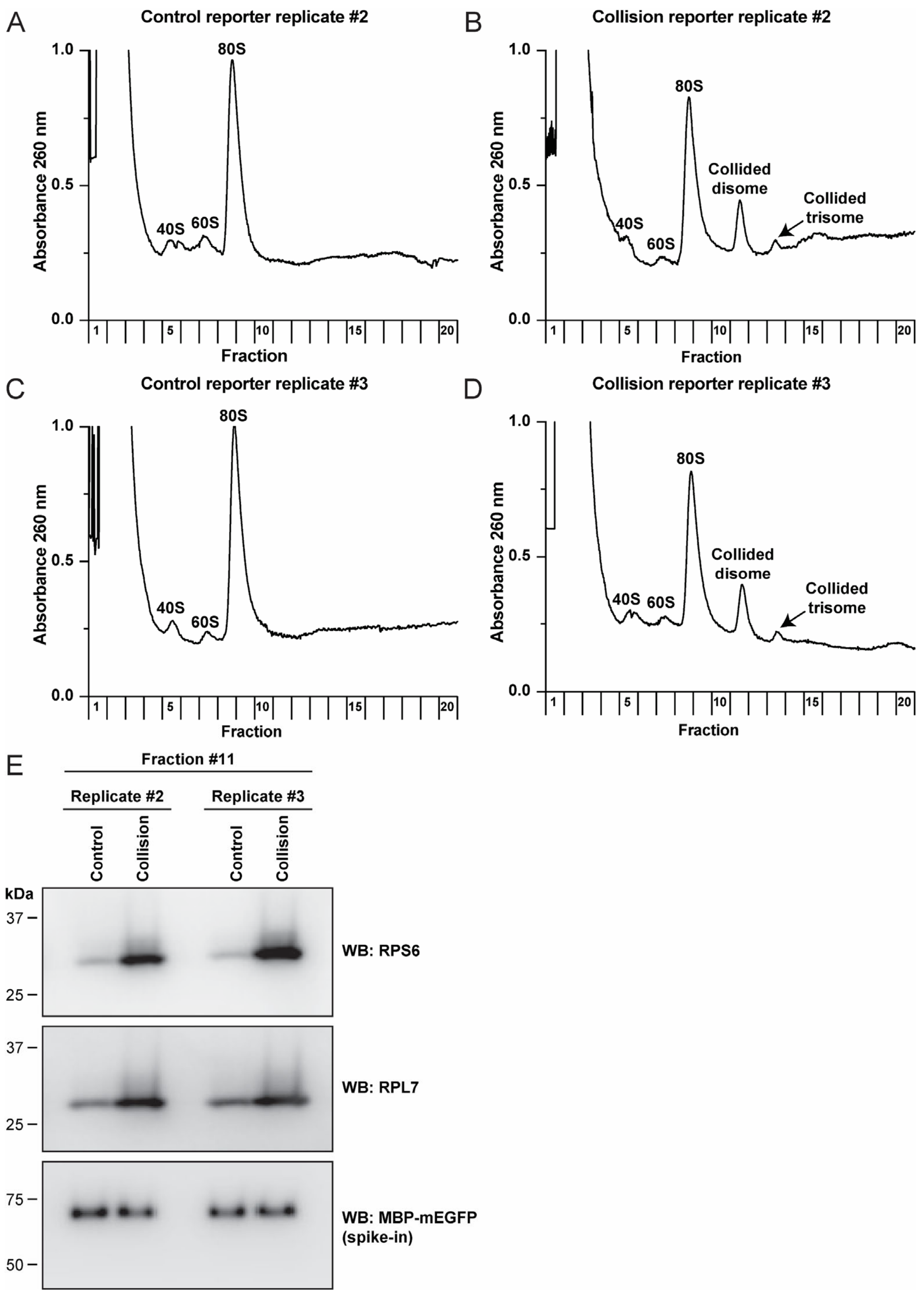

Figure S2. The collision reporter mRNA, but not the control reporter mRNA, generates nuclease-resistant collided ribosomes. A-D) Replicates for polysome analysis of translated control and collision reporter mRNAs with nuclease treatment related to Figure 2. The collision reporter mRNA generates nuclease-resistant collided disomes and trisomes, with a concurrent decrease in monosomes as compared to the control reporter. E) Anti-RPS6 and anti-RPL7 Western blots of fraction #11 that contains disomes. Recombinant MBP-mEGFP was spiked in and used as a loading control.

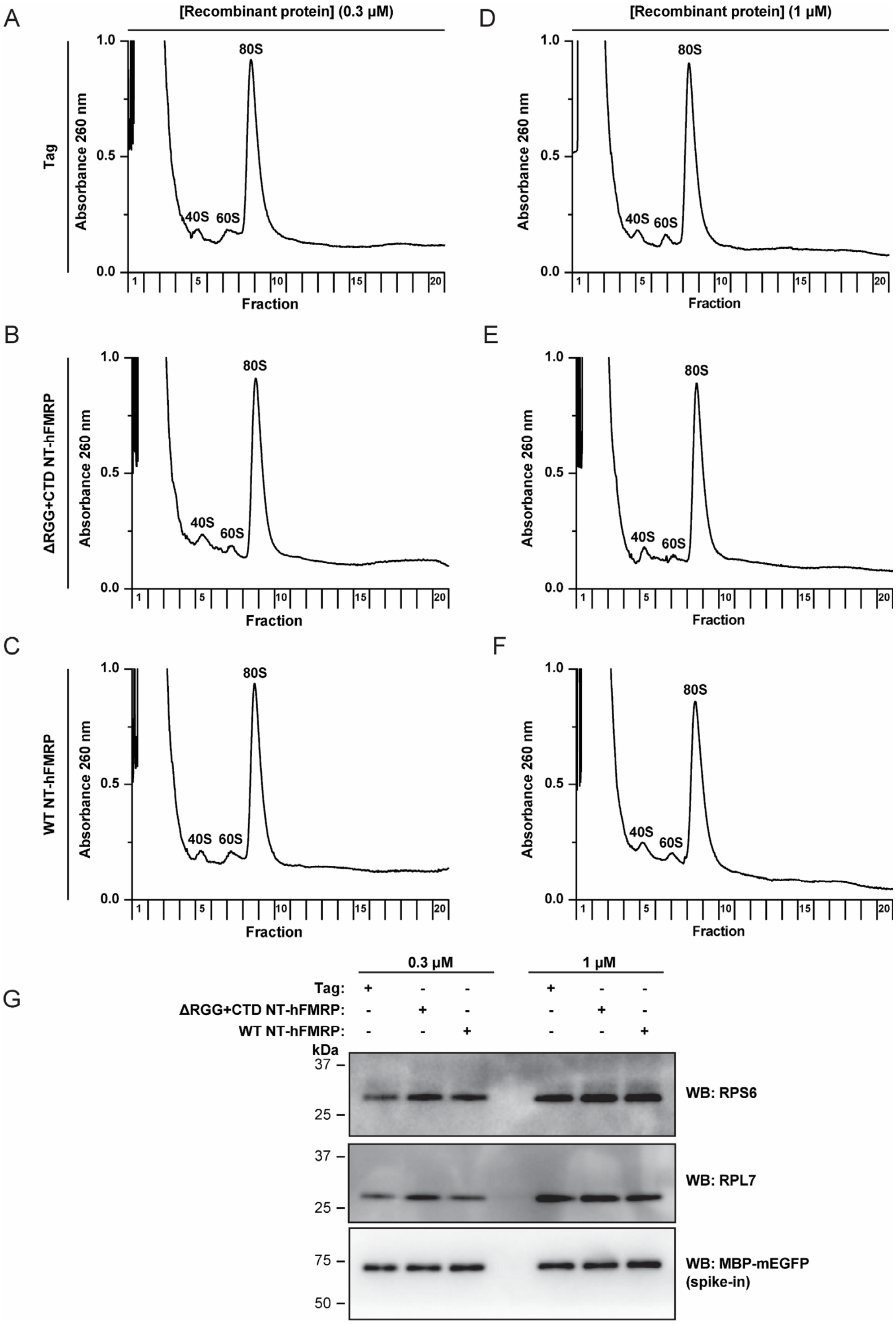

Figure S3. WT NT-hFMRP does not cause detectable nuclease-resistant ribosome collisions on nLuc mRNA in RRL. A-C) Polysome analysis of *in vitro* translation reactions with nuclease treatment and the indicated recombinant proteins at 0.3 μM final (A-C) or 1 μM final (D-F). G) Anti-RPS6 and anti-RPL7 Western blots of fraction #11 that contains disomes. Recombinant MBP-mEGFP was spiked in and used as a loading control.

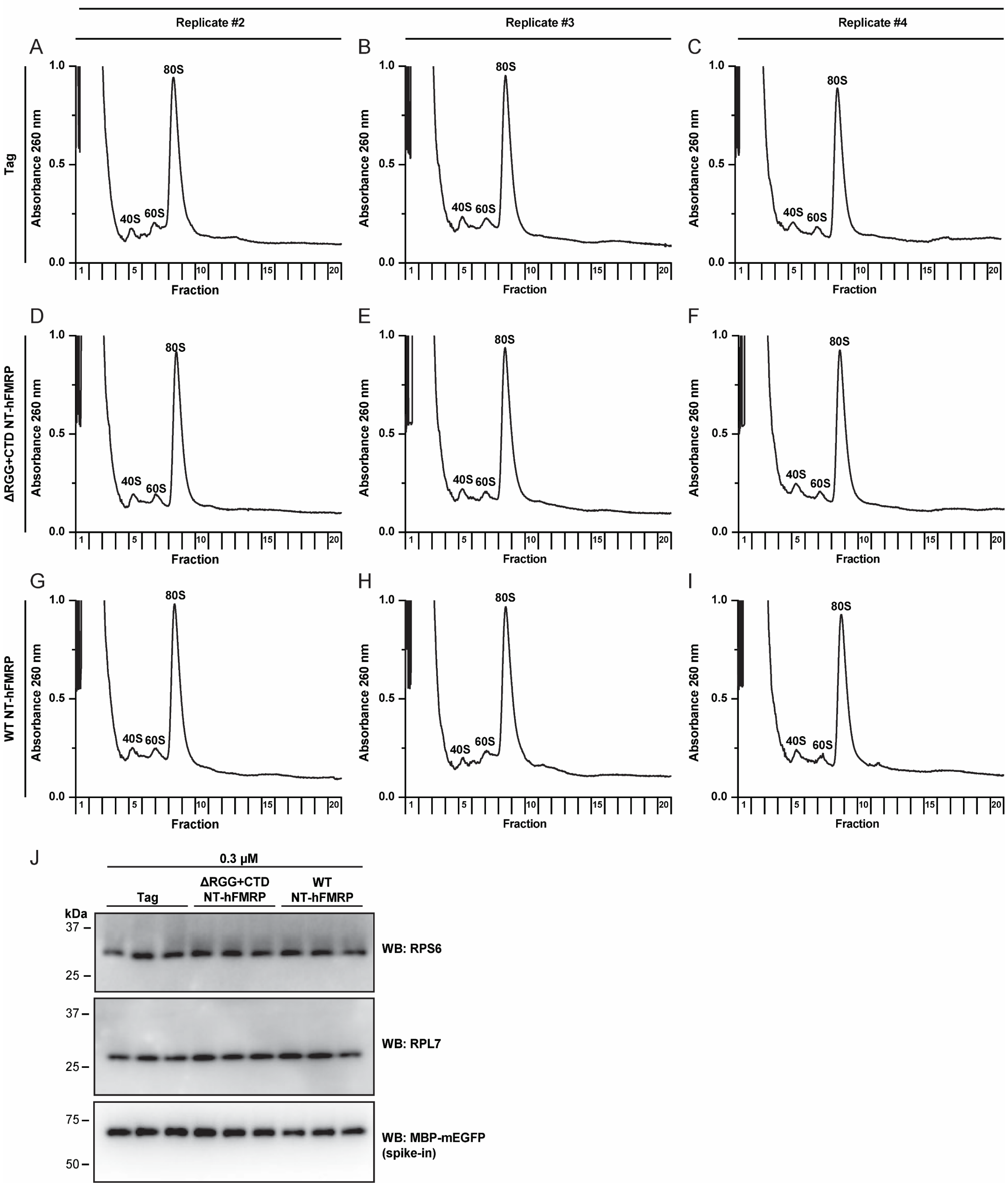

Figure S4. WT NT-hFMRP does not cause detectable nuclease-resistant ribosome collisions in RRL at 0.3 µM on nLuc mRNA. A-I) Replicates of polysome analysis of *in vitro* translation reactions with nuclease treatment and the indicated recombinant proteins at 0.3 µM final related to Figure S3. J) Anti-RPS6 and anti-RPL7 Western blots of fraction #11 that contains disomes. Recombinant MBP-mEGFP was spiked in and used as a loading control.

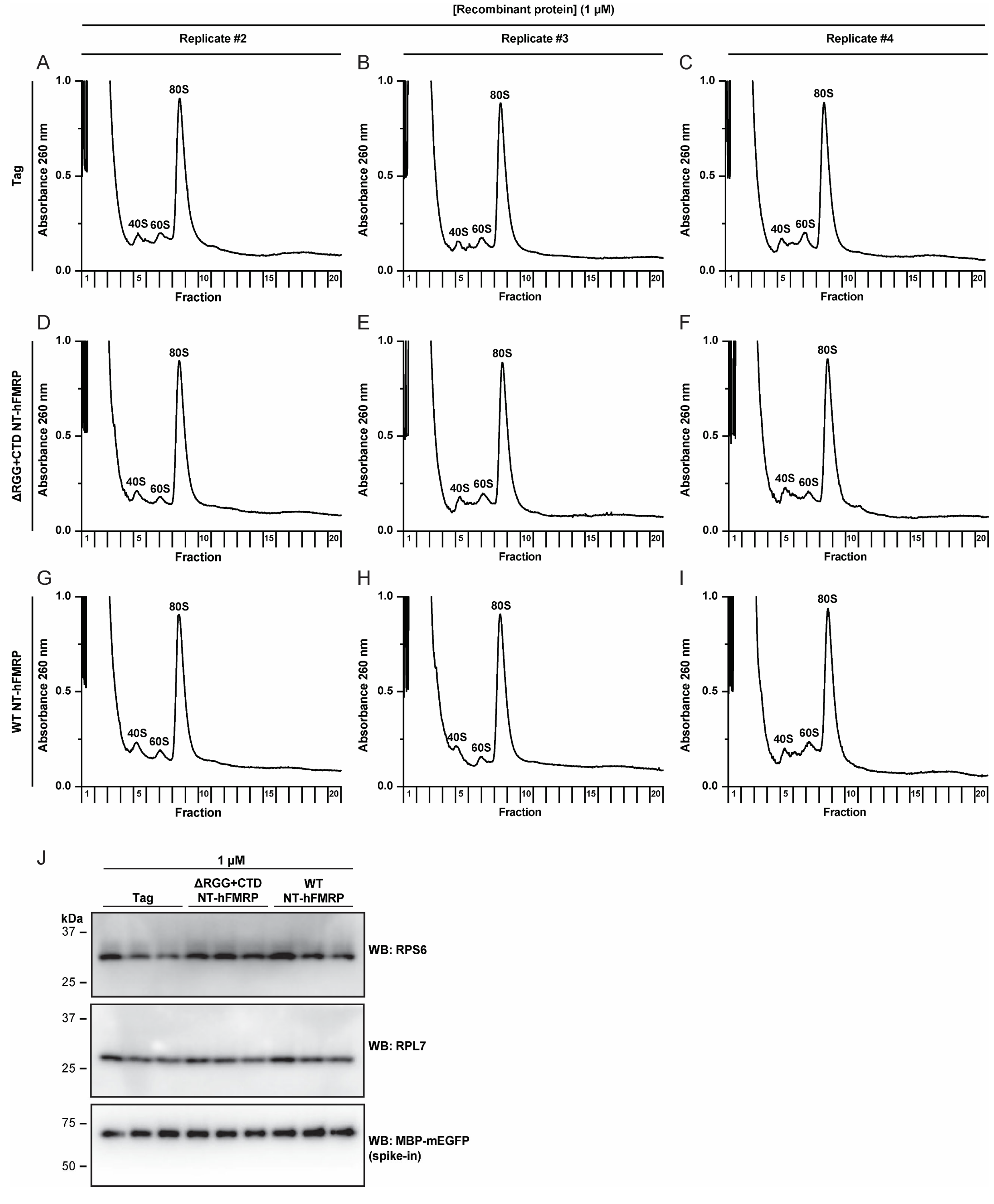

Figure S5. WT NT-hFMRP does not cause detectable nuclease-resistant ribosome collisions in RRL at 1 µM on nLuc mRNA. A-I) Replicates of polysome analysis of *in vitro* translation reactions with nuclease treatment and the indicated recombinant proteins at 1 µM final related to Figure S3. J) Anti-RPS6 and anti-RPL7 Western blots of fraction #11 that contains disomes. Recombinant MBP-mEGFP was spiked in and used as a loading control.

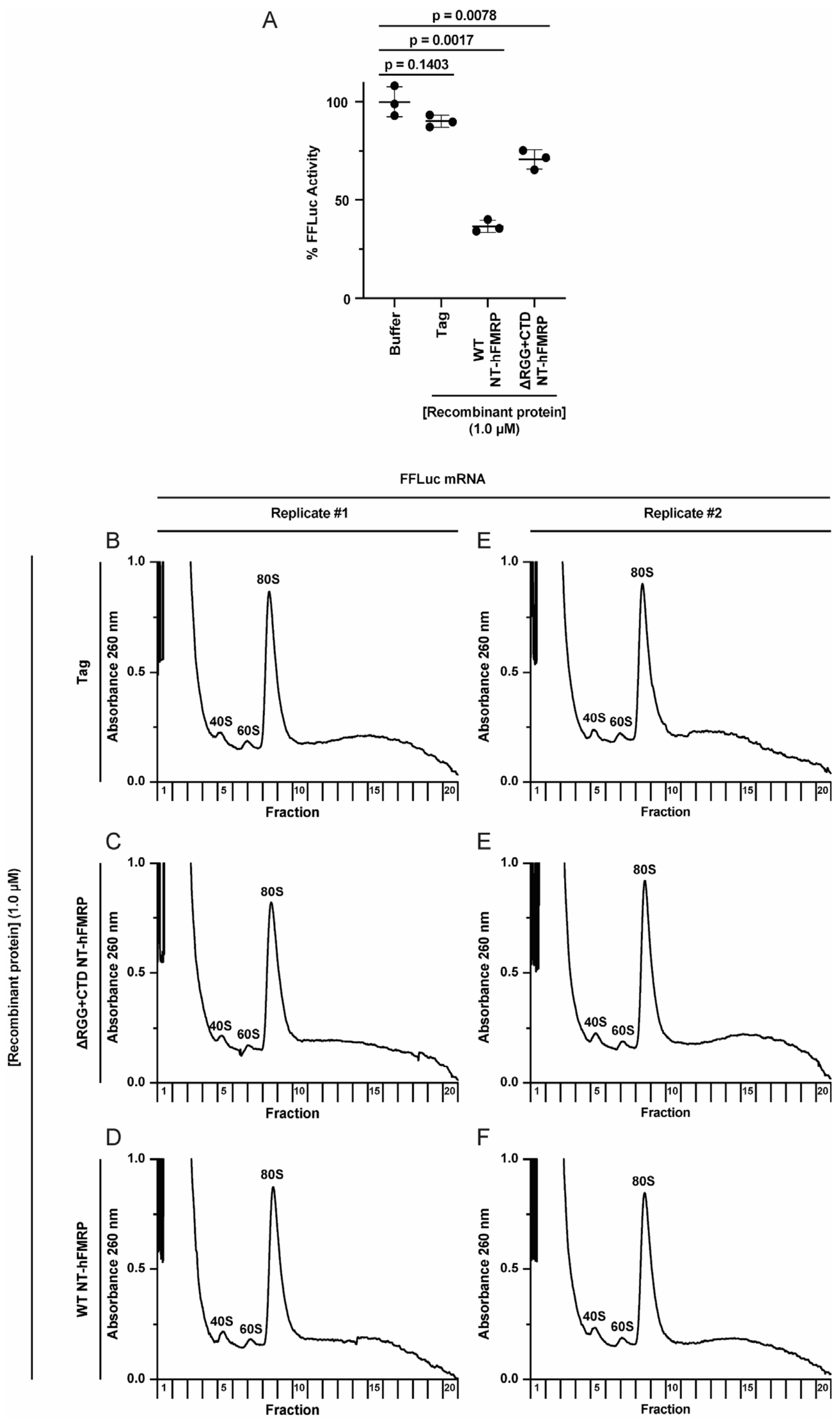

Figure S6. WT NT-hFMRP does not cause detectable nuclease-resistant ribosome collisions in RRL at 1 μM on FFLuc mRNA. A) *In vitro* translation of FFLuc reporter mRNAs pre-incubated with protein storage buffer (Buffer) or the indicated recombinant protein (1 μM final). Data are shown as mean ± SD. n = 3 biological replicates. Comparisons were made using a two-tailed unpaired t test with Welch's correction. B-F) Polysome analysis of *in vitro* translation reactions with nuclease treatment and the indicated recombinant proteins at 1 μM final. Duplicate samples are shown.

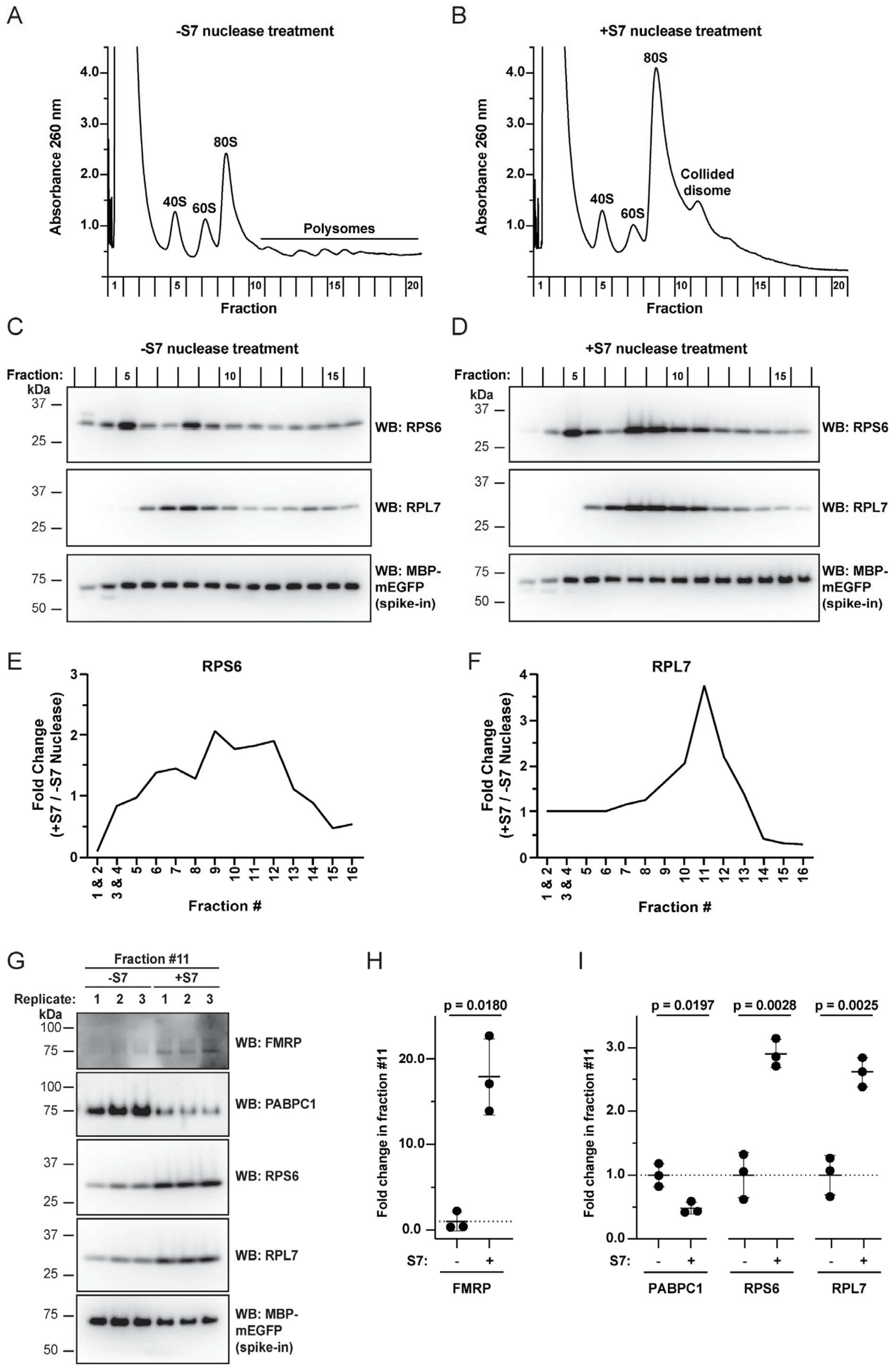

Figure S7. FMRP co-sediments with nuclease-resistant disomes from cells. A-B) Polysome analysis of N2A cell lysates without (A) and with (B) S7 nuclease treatment. C-D) Anti-RPS6 and anti-RPL7 Western blots of fractions 1-16 of untreated (C) or nuclease-treated cell lysates (D). The first lane contains fractions 1 & 2 combined and the second lane contains fractions 3 & 4 combined. The third lane and on contain single fractions. Recombinant MBP-mEGFP was spiked-in and used as a loading control. Western blots between the two conditions

were treated identically (i.e., gels were run in parallel with primary antibodies dilutions, secondary antibody dilutions, & ECL chemiluminescence regent were made in batch and split equally, and blots were imaged for the same amount of time in parallel). E-F) Fold change of RPS6 (E) and RPL7 (F) levels in each fraction with and without S7 nuclease treatment from panels C & D. Signal from each fraction was normalized to their respective MBP-mEGFP spike-in control and then compared between the two conditions. G) Anti-FMRP, anti-PABP, anti-RPS6, and anti-RPL7 Western blot analysis of fraction #11 that contains disomes. Recombinant MBP-mEGFP was spiked-in and used as a loading control. H-I) Quantification of the indicated proteins in panel G. Bands were first normalized to MBP-mEGFP and then set relative to untreated. Data are shown as mean ± SD. n = 3 biological replicates. Comparisons were made using a two-tailed unpaired t test with Welch's correction.

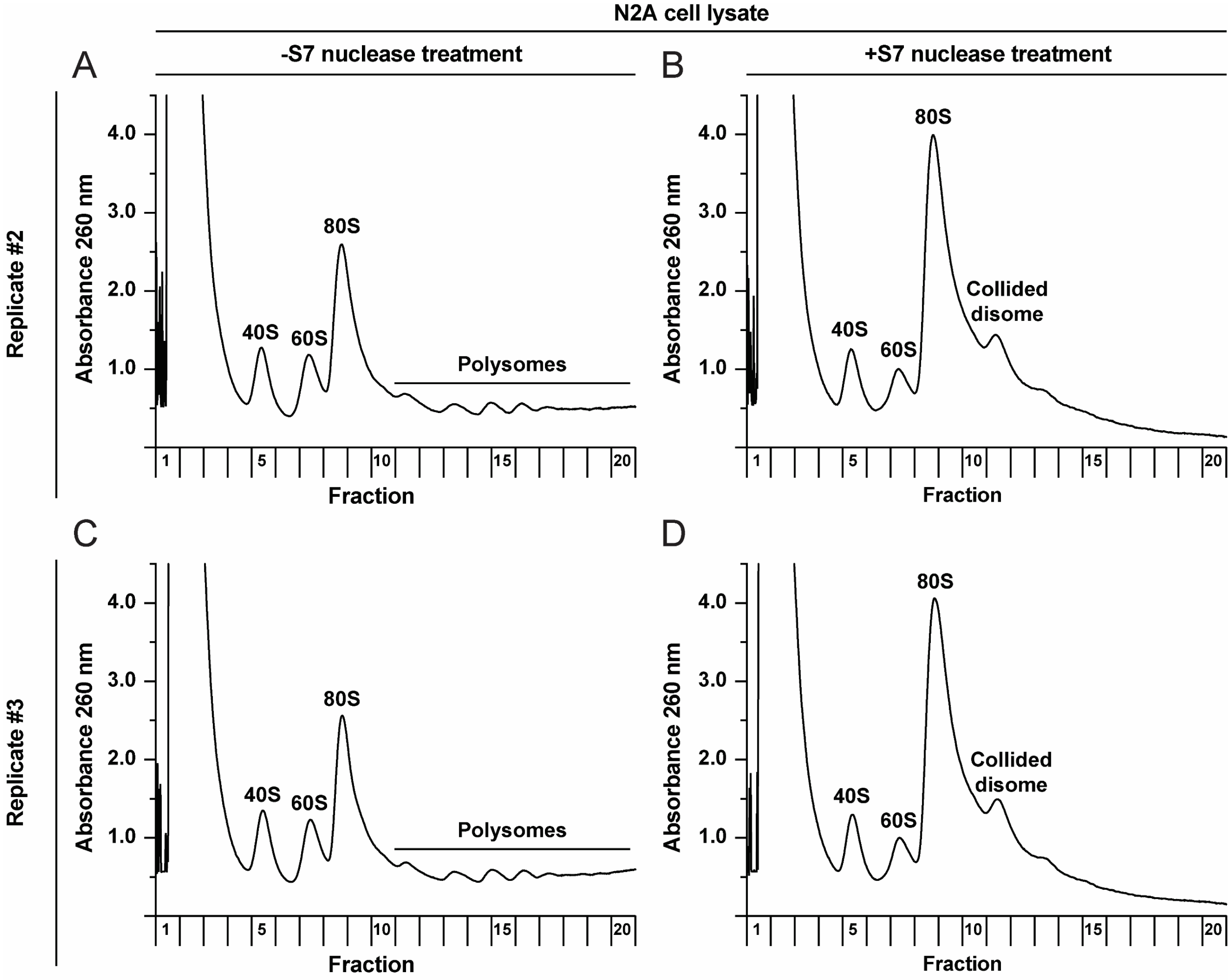

Figure S8. N2A cells naturally have nuclease-resistant disomes. A-D) Replicates of polysome analysis of N2A cell lysates without (A & C) and with (B & D) S7 nuclease treatment related to Figure S7.

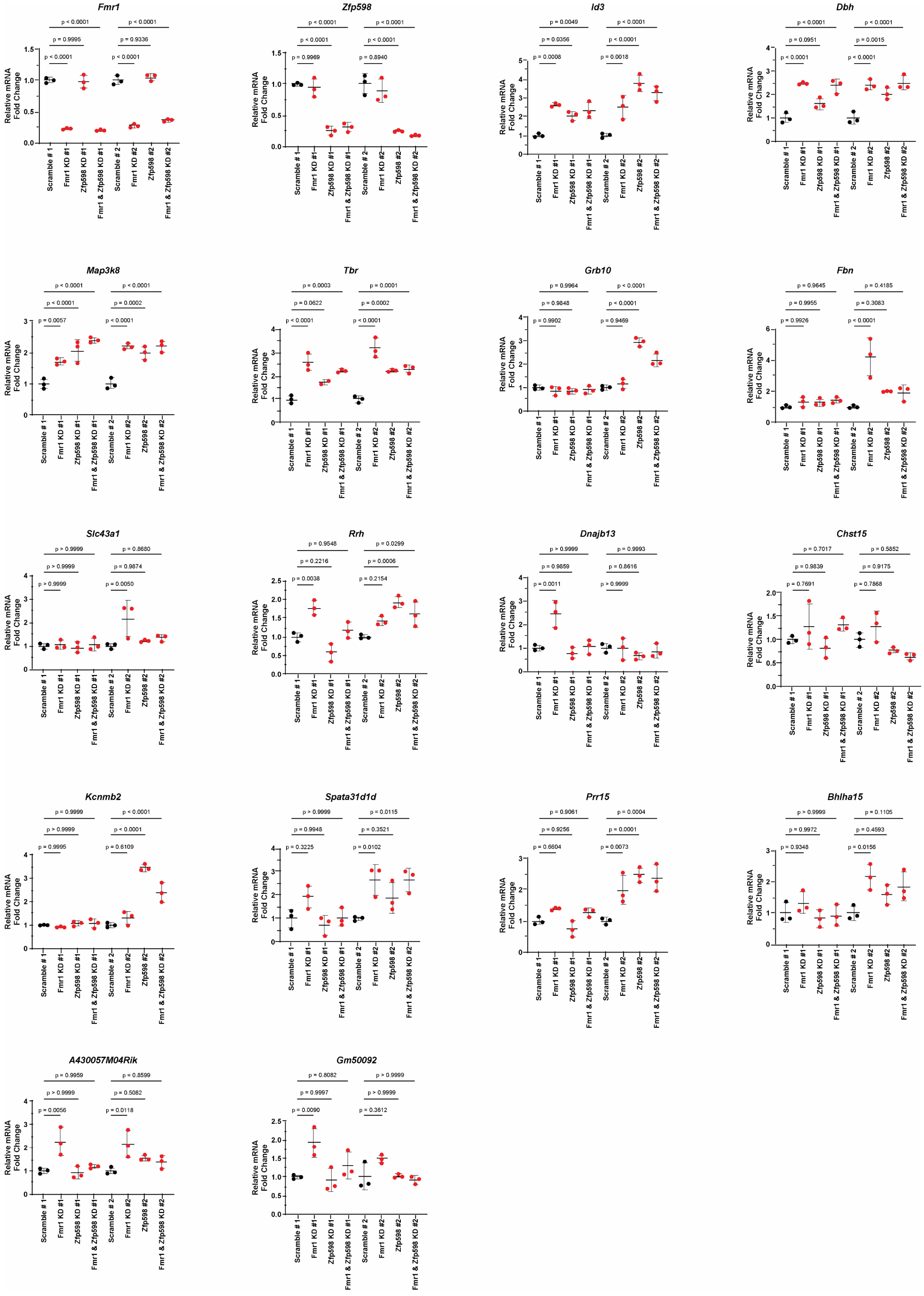

Figure S9. RT-qPCR validation of the putative FMRP-mediated NGD targets identified by RNA-seq. 72 hr knockdowns were repeated with two independent siRNAs (labeled #1 and #2),

individually or in combination as indicated. Steady state mRNA levels were measured by RT-qPCR with GAPDH as the reference gene. Only *Id3, Dbh, Map3k8, and Tbr1* were reproducible by RT-qPCR with both independent siRNAs. Double knockdown did not result in increased levels over single knockdowns, suggesting that FMRP and ZFP598 act in the same decay pathway for *Id3, Dbh, Map3k8, and Tbr1*. Data are shown as mean ± SD. n = 3 biological replicates. Comparisons were made using an ordinary one-way ANOVA with Tukey's multiple comparisons.

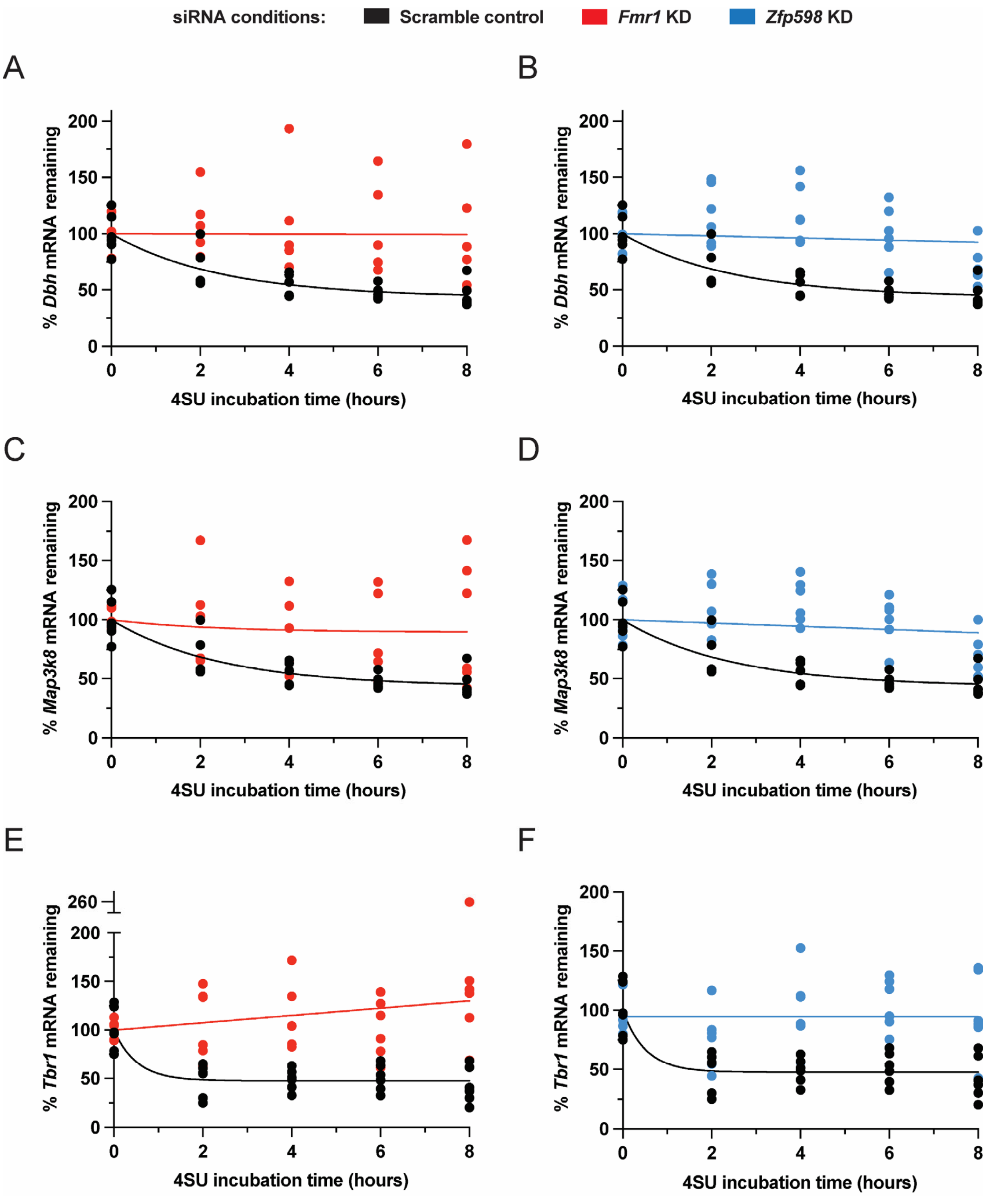

Figure S10. *Dbh*, *Rrh*, and *Tbr1* mRNAs are stabilized upon *Fmr1* and *Zfp598* KD compared to scramble control conditions in N2A cells. A-F) Roadblock-qPCR was used to measure mRNA half-lives ($t_{1/2}$) of *Dbh* mRNA (A, B), *Rrh* mRNA (C, D), and *Tbr1* mRNA (E, F) in N2A cells. The Scramble negative control is shown in black (and is the same in A & B, in C & D, and in E & F), *Fmr1* KD in red, and *Zfp598* KD in blue. n=6 biological replicates. One phase decay trend lines calculated by nonlinear regression are shown. However, due to the mRNA levels not decreasing below 50% during the 8 hr time course, the $t_{1/2}$ for these mRNAs could not be determined. Nevertheless, in all three examples, the one phase decay trend lines for both *Fmr1* KD and *Zfp598* KD are markedly above the Scramble negative control.

Reporter Sequences

T7 promoter
Human beta globin 5' UTR
Heterologous 5' UTR
Start codon
Reporter Sequence (nLuc or FFLuc)
Hairpin (HP)
Stop codon

Control nLuc reporter
*TAATACGACTCACTATAG*GGACATTTGCTTCTGACACAACTGTGTTCACTAGCAACCTCAAA
CAGACACCATGGTGTTTACCCTTGAAGATTTTGTCGGCGATTGGCGCCAGACCGCGCGCT
ATAACCTCGATCAAGTGCTCGAACAAGGCGGCGTGAGCAGCCTGTTTCAGAACCTGGGCG
TGAGCGTGACCCCGATTCAGCGCATTGTGCTGAGCGGCGAAAACGGCCTGAAAATTGATA
TTCATGTGATTATTCCGTATGAAGGCCTGAGCGGCGATCAGATGGGCCAGATTGAAAAAAT
TTTTAAAGTCGTATATCCTGTCGATGATCATCATTTTAAAGTGATTCTGCATTATGGCACCCT
GGTGATTGATGGCGTGACCCCGAACATGATTGATTATTTTGGCCGCCCGTATGAAGGCATT
GCTGTTTTTGATGGCAAGAAGATTACCGTGACCGGCACCCTGTGGAACGGCAACAAAATTA
TTGATGAACGCCTGATTAACCCAGATGGCAGCCTGCTGTTTCGCGTGACCATTAACGGCGT
GACCGGCTGGCGCCTGTGCGAACGCATTCTCGCATAA GGCCGCGACTCTAGA

Collision nLuc reporter
*TAATACGACTCACTATAG*GGACATTTGCTTCTGACACAACTGTGTTCACTAGCAACCTCAAA
CAGACACCATGGTGTTTACCCTTGAAGATTTTGTCGGCGATTGGCGCCAGACCGCGCGCT
ATAACCTCGATCAAGTGCTCGAACAAGGCGGCGTGAGCAGCCTGTTTCAGAACCTGGGCG
TGAGCGTGACCCCGATTCAGCGCATTGTGCTGAGCGGCGAAAACGGCCTGAAAATTGATA
TTCATGTGATTATTCCGTATGAAGGCCTGAGCGGCGATCAGATGGGCCAGATTGAAAAAAT
TTTTAAAGTCGTATATCCTGTCGATGATCATCATTTTAAAGTGATTCTGCATTATGGCACCCT
GGTGATTGATGGCGTGACCCCGAACATGATTGATTATTTTGGCCGCCCGTATGAAGGCATT
GCTGTTTTTGATGGCAAGAAGATTACCGTGACCGGCACCCTGTGGAACGGCAACAAAATTA
TTGATGAACGCCTGATTAACCCAGATGGCAGCCTGCTGTTTCGCGTGACCATTAACGGCGT
GACCGGCTGGCGCCTGTGCGAACGCATTCTCGCACAACAACAACAACAATTGGGGCGCGT
GGTGGCGGCTGCAGCCGCCACCACGCGCCCCGGCAACAACAACAACAATCTAGA

FFLuc reporter
*TAATACGACTCACTATAG*GGCGAATTGGGCCCTCTAGATGCATGCTCGAGCGGCC
GCCAGTGTGATGGATATCTGCAGAATTCGGCTTCACCATGGAAGATGCCAAAAACA
TTAAGAAGGGCCCAGCGCCATTCTACCCACTCGAAGACGGGACCGCCGGCGAGC
AGCTGCACAAAGCCATGAAGCGCTACGCCCTGGTGCCCGGCACCATCGCCTTTAC
CGACGCACATATCGAGGTGGACATTACCTACGCCGAGTACTTCGAGATGAGCGTT
CGGCTGGCAGAAGCTATGAAGCGCTATGGGCTGAATACAAACCATCGGATCGTGG
TGTGCAGCGAGAATAGCTTGCAGTTCTTCATGCCCGTGTTGGGTGCCCTGTTCATC
GGTGTGGCTGTGGCCCCAGCTAACGACATCTACAACGAGCGCGAGCTGCTGAACA
GCATGGGCATCAGCCAGCCCACCGTCGTATTCGTGAGCAAGAAAGGGCTGCAAAA
GATCCTCAACGTGCAAAAGAAGCTACCGATCATACAAAAGATCATCATCATGGATA
GCAAGACCGACTACCAGGGCTTCCAAAGCATGTACACCTTCGTGACTTCCCATTTG
CCACCCGGCTTCAACGAGTACGACTTCGTGCCCGAGAGCTTCGACCGGGACAAAA
CCATCGCCCTGATCATGAACAGTAGTGGCAGTACCGGATTGCCCAAGGGCGTAGC

CCTACCGCACCGCACCGCTTGTGTCCGATTCAGTCATGCCCGCGACCCCATCTTC
GGCAACCAGATCATCCCCGACACCGCTATCCTCAGCGTGGTGCCATTTCACCACG
GCTTCGGCATGTTCACCACGCTGGGCTACTTGATCTGCGGCTTTCGGGTCGTGCT
CATGTACCGCTTCGAGGAGGAGCTATTCTTGCGCAGCTTGCAAGACTATAAGATTC
AATCTGCCCTGCTGGTGCCCACACTATTTAGCTTCTTCGCTAAGAGCACTCTCATC
GACAAGTACGACCTAAGCAACTTGCACGAGATCGCCAGCGGCGGGGCGCCGCTC
AGCAAGGAGGTAGGTGAGGCCGTGGCCAAACGCTTCCACCTACCAGGCATCCGC
CAGGGCTACGGCCTGACAGAAACAACCAGCGCCATTCTGATCACCCCCGAAGGG
GACGACAAGCCTGGCGCAGTAGGCAAGGTGGTGCCCTTCTTCGAGGCTAAGGTG
GTGGACTTGGACACCGGTAAGACACTGGGTGTGAACCAGCGCGGCGAGCTGTGC
GTCCGTGGCCCCATGATCATGAGCGGCTACGTTAACAACCCCGAGGCTACAAACG
CTCTCATCGACAAGGACGGCTGGCTGCACAGCGGCGACATCGCCTACTGGGACG
AGGACGAGCACTTCTTCATCGTGGACCGGCTGAAGAGCCTGATCAAATACAAGGG
CTACCAGGTAGCCCCAGCCGAACTGGAGAGCATCCTGCTGCAACACCCCAACATC
TTCGACGCCGGGGTCGCCGGCCTGCCCGACGACGATGCCGGCGAGCTGCCCGC
CGCAGTCGTCGTGCTGGAACACGGTAAAACCATGACCGAGAAGGAGATCGTGGAC
TATGTGGCCAGCCAGGTTACAACCGCCAAGAAGCTGCGCGGTGGTGTTGTGTTCG
TGGACGAGGTGCCTAAAGGACTGACCGGCAAGTTGGACGCCCGCAAGATCCGCG
AGATTCTCATTAAGGCCAAGAAGGGCGGCAAGATCGCCGTGTAAAAGCCGAATTC
CAGCACACTGGCGGCCGTTACTAGTGGATCCGAGCTCGGTACCAAGCTT

Recombinant Protein Coding Sequences

Start codon
MBP
His6
NT-hFMRP (WT and mutant)
Stop codon

His6-MBP

ATGGGTTCTTCTCACCATCACCATCACCATGGTTCTTCTATGAAAATCGAAGAAGGTAAACT
GGTAATCTGGATTAACGGCGATAAAGGCTATAACGGTCTCGCTGAAGTCGGTAAGAAATTC
GAGAAAGATACCGGAATTAAAGTCACCGTTGAGCATCCGGATAAACTGGAAGAGAAATTCC
CACAGGTTGCGGCAACTGGCGATGGCCCTGACATTATCTTCTGGGCACACGACCGCTTTG
GTGGCTACGCTCAATCTGGCCTGTTGGCTGAAATCACCCCGGACAAAGCGTTCCAGGACA
AGCTGTATCCGTTTACCTGGGATGCCGTACGTTACAACGGCAAGCTGATTGCTTACCCGAT
CGCTGTTGAAGCGTTATCGCTGATTTATAACAAAGATCTGCTGCCGAACCCGCCAAAAACC
TGGGAAGAGATCCCGGCGCTGGATAAAGAACTGAAAGCGAAAGGTAAGAGCGCGCTGAT
GTTCAACCTGCAAGAACCGTACTTCACCTGGCCGCTGATTGCTGCTGACGGGGGTTATGC
GTTCAAGTATGAAAACGGCAAGTACGACATTAAAGACGTGGGCGTGGATAACGCTGGCGC
GAAAGCGGGTCTGACCTTCCTGGTTGACCTGATTAAAAACAAACACATGAATGCAGACACC
GATTACTCCATCGCAGAAGCTGCCTTTAATAAAGGCGAAACAGCGATGACCATCAACGGCC
CGTGGGCATGGTCCAACATCGACACCAGCAAAGTGAATTATGGTGTAACGGTACTGCCGA
CCTTCAAGGGTCAACCATCCAAACCGTTCGTTGGCGTGCTGAGCGCAGGTATTAACGCCG
CCAGTCCGAACAAAGAGCTGGCAAAAGAGTTCCTCGAAAACTATCTGCTGACTGATGAAGG
TCTGGAAGCGGTTAATAAAGACAAACCGCTGGGTGCCGTAGCGCTGAAGTCTTACGAGGA
AGAGTTGGCGAAAGATCCACGTATTGCCGCCACTATGGAAAACGCCCAGAAAGGTGAAAT
CATGCCGAACATCCCGCAGATGTCCGCTTTCTGGTATGCCGTGCGTACTGCGGTGATCAA
CGCCGCCAGCGGTCGTCAGACTGTCGATGAAGCCCTGAAAGACGCGCAGACTAATGGGA
TCGAGGAAAACCTGTACTTCCAATCCAATATTGGAAGTGGATAA

MBP-(WT NT-hFMRP)-His6

ATGGGTTCTTCTATGAAAATCGAAGAAGGTAAACTGGTAATCTGGATTAACGGCGATAAAG
GCTATAACGGTCTCGCTGAAGTCGGTAAGAAATTCGAGAAAGATACCGGAATTAAAGTCAC
CGTTGAGCATCCGGATAAACTGGAAGAGAAATTCCCACAGGTTGCGGCAACTGGCGATGG
CCCTGACATTATCTTCTGGGCACACGACCGCTTTGGTGGCTACGCTCAATCTGGCCTGTTG
GCTGAAATCACCCCGGACAAAGCGTTCCAGGACAAGCTGTATCCGTTTACCTGGGATGCC
GTACGTTACAACGGCAAGCTGATTGCTTACCCGATCGCTGTTGAAGCGTTATCGCTGATTT
ATAACAAAGATCTGCTGCCGAACCCGCCAAAAACCTGGGAAGAGATCCCGGCGCTGGATA
AAGAACTGAAAGCGAAAGGTAAGAGCGCGCTGATGTTCAACCTGCAAGAACCGTACTTCA
CCTGGCCGCTGATTGCTGCTGACGGGGGTTATGCGTTCAAGTATGAAAACGGCAAGTACG
ACATTAAAGACGTGGGCGTGGATAACGCTGGCGCGAAAGCGGGTCTGACCTTCCTGGTTG
ACCTGATTAAAAACAAACACATGAATGCAGACACCGATTACTCCATCGCAGAAGCTGCCTT
TAATAAAGGCGAAACAGCGATGACCATCAACGGCCCGTGGGCATGGTCCAACATCGACAC
CAGCAAAGTGAATTATGGTGTAACGGTACTGCCGACCTTCAAGGGTCAACCATCCAAACCG
TTCGTTGGCGTGCTGAGCGCAGGTATTAACGCCGCCAGTCCGAACAAAGAGCTGGCAAAA
GAGTTCCTCGAAAACTATCTGCTGACTGATGAAGGTCTGGAAGCGGTTAATAAAGACAAAC
CGCTGGGTGCCGTAGCGCTGAAGTCTTACGAGGAAGAGTTGGCGAAAGATCCACGTATTG
CCGCCACTATGGAAAACGCCCAGAAAGGTGAAATCATGCCGAACATCCCGCAGATGTCCG
CTTTCTGGTATGCCGTGCGTACTGCGGTGATCAACGCCGCCAGCGGTCGTCAGACTGTCG
ATGAAGCCCTGAAAGACGCGCAGACTAATGGGATCGAGGAAAACCTGTACTTCCAATCCAA
TGCACGCTTCCACGAACAATTTATTGTTCGCGAGGACCTGATGGGCTTGGCGATTGGCAC
CCACGGCGCGAACATTCAGCAGGCGCGTAAAGTCCCAGGTGTTACCGCTATTGATCTGGA

CGAAGACACGTGCACCTTTCATATTTATGGTGAAGACCAGGATGCTGTGAAAAAGGCGCGT
TCCTTCCTGGAATTTGCGGAAGACGTGATCCAGGTTCCGCGTAACCTGGTTGGTAAAGTCA
TCGGCAAAAACGGTAAGTTAATCCAAGAGATCGTGGACAAAAGCGGGGTAGTTCGCGTTC
GGATTGAAGCGGAAAATGAGAAGAACGTTCCGCAGGAGGAAGAAATTATGCCGCCAAATA
GCCTGCCGAGCAACAACTCACGTGTCGGTCCGAACGCTCCGGAAGAGAAGAAGCACCTG
GATATTAAAGAGAACAGCACCCATTTCAGCCAACCAAACTCCACTAAGGTGCAGCGTGTTT
TGGTAGCCAGCTCCGTTGTTGCCGGTGAGTCGCAAAAGCCGGAACTGAAAGCGTGGCAG
GGTATGGTGCCGTTCGTCTTTGTGGGCACCAAGGACAGCATCGCCAACGCAACGGTTCTG
CTGGACTACCATCTGAATTACCTGAAAGAAGTCGATCAGCTTCGTTTGGAACGCTTGCAAA
TCGATGAGCAACTGCGCCAGATCGGTGCGAGCTCTCGCCCGTCTCCGAACCGTACCGACA
AAGAGAAGAGCTACGTGACCGACGACGGTCAAGGTATGGGCCGTGGCAGCCGTCCGTAT
CGTAATAGGGGACATGGCCGCCGTGGTCCGGGTTACACCTCAGGTACGAACTCTGAGGC
GTCTAACGCCTCCGAAACCGAGTCGGATCATCGTGATGAGCTGAGCGACTGGTCACTGGC
GCCGACCGAAGAGGAGCGCGAGAGCTTTCTGCGTCGCGGTGATGGTCGCCGCAGAGGC
GGTGGCGGTCGTGGCCAGGGCGGCCGTGGCAGAGGCGGCGGTTTTAAAGGTAATGATGA
TCACAGCCGCACTGACAACCGTCCGCGTAATCCGCGTGAGGCGAAGGGCAGAACTACAG
ATGGTAGCTTGCAAATCCGTGTGGACTGTAATAACGAACGCAGCGTGCATACGAAAACCCT
ACAAAACACCAGTAGCGAGGGTAGCCGCCTTCGTACCGGTAAAGACCGCAACCAGAAGAA
AGAGAAACCGGACAGCGTTGATGGCCAACAACCGTTGGTGAATGGTGTTCCGGGTTCTTC
TCACCATCACCATCACCATTAA

MBP-(I304N NT-hFMRP)-His6

ATGGGTTCTTCTATGAAAATCGAAGAAGGTAAACTGGTAATCTGGATTAACGGCGATAAAG
GCTATAACGGTCTCGCTGAAGTCGGTAAGAAATTCGAGAAAGATACCGGAATTAAAGTCAC
CGTTGAGCATCCGGATAAACTGGAAGAGAAATTCCCACAGGTTGCGGCAACTGGCGATGG
CCCTGACATTATCTTCTGGGCACACGACCGCTTTGGTGGCTACGCTCAATCTGGCCTGTTG
GCTGAAATCACCCCGGACAAAGCGTTCCAGGACAAGCTGTATCCGTTTACCTGGGATGCC
GTACGTTACAACGGCAAGCTGATTGCTTACCCGATCGCTGTTGAAGCGTTATCGCTGATTT
ATAACAAAGATCTGCTGCCGAACCCGCCAAAAACCTGGGAAGAGATCCCGGCGCTGGATA
AAGAACTGAAAGCGAAAGGTAAGAGCGCGCTGATGTTCAACCTGCAAGAACCGTACTTCA
CCTGGCCGCTGATTGCTGCTGACGGGGGTTATGCGTTCAAGTATGAAAACGGCAAGTACG
ACATTAAAGACGTGGGCGTGGATAACGCTGGCGCGAAAGCGGGTCTGACCTTCCTGGTTG
ACCTGATTAAAAACAAACACATGAATGCAGACACCGATTACTCCATCGCAGAAGCTGCCTT
TAATAAAGGCGAAACAGCGATGACCATCAACGGCCCGTGGGCATGGTCCAACATCGACAC
CAGCAAAGTGAATTATGGTGTAACGGTACTGCCGACCTTCAAGGGTCAACCATCCAAACCG
TTCGTTGGCGTGCTGAGCGCAGGTATTAACGCCGCCAGTCCGAACAAAGAGCTGGCAAAA
GAGTTCCTCGAAAACTATCTGCTGACTGATGAAGGTCTGGAAGCGGTTAATAAAGACAAAC
CGCTGGGTGCCGTAGCGCTGAAGTCTTACGAGGAAGAGTTGGCGAAAGATCCACGTATTG
CCGCCACTATGGAAAACGCCCAGAAAGGTGAAATCATGCCGAACATCCCGCAGATGTCCG
CTTTCTGGTATGCCGTGCGTACTGCGGTGATCAACGCCGCCAGCGGTCGTCAGACTGTCG
ATGAAGCCCTGAAAGACGCGCAGACTAATGGGATCGAGGAAAACCTGTACTTCCAATCCAA
TGCACGCTTCCACGAACAATTTATTGTTCGCGAGGACCTGATGGGCTTGGCGATTGGCAC
CCACGGCGCGAACATTCAGCAGGCGCGTAAAGTCCCAGGTGTTACCGCTATTGATCTGGA
CGAAGACACGTGCACCTTTCATATTTATGGTGAAGACCAGGATGCTGTGAAAAAGGCGCGT
TCCTTCCTGGAATTTGCGGAAGACGTGATCCAGGTTCCGCGTAACCTGGTTGGTAAAGTCA
TCGGCAAAAACGGTAAGTTAAACCAAGAGATCGTGGACAAAAGCGGGGTAGTTCGCGTTC
GGATTGAAGCGGAAAATGAGAAGAACGTTCCGCAGGAGGAAGAAATTATGCCGCCAAATA
GCCTGCCGAGCAACAACTCACGTGTCGGTCCGAACGCTCCGGAAGAGAAGAAGCACCTG
GATATTAAAGAGAACAGCACCCATTTCAGCCAACCAAACTCCACTAAGGTGCAGCGTGTTT
TGGTAGCCAGCTCCGTTGTTGCCGGTGAGTCGCAAAAGCCGGAACTGAAAGCGTGGCAG
GGTATGGTGCCGTTCGTCTTTGTGGGCACCAAGGACAGCATCGCCAACGCAACGGTTCTG

CTGGACTACCATCTGAATTACCTGAAAGAAGTCGATCAGCTTCGTTTGGAACGCTTGCAAA
TCGATGAGCAACTGCGCCAGATCGGTGCGAGCTCTCGCCCGTCTCCGAACCGTACCGACA
AAGAGAAGAGCTACGTGACCGACGACGGTCAAGGTATGGGCCGTGGCAGCCGTCCGTAT
CGTAATAGGGGACATGGCCGCCGTGGTCCGGGTTACACCTCAGGTACGAACTCTGAGGC
GTCTAACGCCTCCGAAACCGAGTCGGATCATCGTGATGAGCTGAGCGACTGGTCACTGGC
GCCGACCGAAGAGGAGCGCGAGAGCTTTCTGCGTCGCGGTGATGGTCGCCGCAGAGGC
GGTGGCGGTCGTGGCCAGGGCGGCCGTGGCAGAGGCGGCGGTTTTAAAGGTAATGATGA
TCACAGCCGCACTGACAACCGTCCGCGTAATCCGCGTGAGGCGAAGGGCAGAACTACAG
ATGGTAGCTTGCAAATCCGTGTGGACTGTAATAACGAACGCAGCGTGCATACGAAAACCCT
ACAAAACACCAGTAGCGAGGGTAGCCGCCTTCGTACCGGTAAAGACCGCAACCAGAAGAA
AGAGAAACCGGACAGCGTTGATGGCCAACAACCGTTGGTGAATGGTGTTCCGGGTTCTTC
TCACCATCACCATCACCATTAA

MBP-(NT-hFMRP ΔRGG+CTD complete)-His6

ATGGGTTCTTCTATGAAAATCGAAGAAGGTAAACTGGTAATCTGGATTAACGGCGATAAAG
GCTATAACGGTCTCGCTGAAGTCGGTAAGAAATTCGAGAAAGATACCGGAATTAAAGTCAC
CGTTGAGCATCCGGATAAACTGGAAGAGAAATTCCCACAGGTTGCGGCAACTGGCGATGG
CCCTGACATTATCTTCTGGGCACACGACCGCTTTGGTGGCTACGCTCAATCTGGCCTGTTG
GCTGAAATCACCCCGGACAAAGCGTTCCAGGACAAGCTGTATCCGTTTACCTGGGATGCC
GTACGTTACAACGGCAAGCTGATTGCTTACCCGATCGCTGTTGAAGCGTTATCGCTGATTT
ATAACAAAGATCTGCTGCCGAACCCGCCAAAAACCTGGGAAGAGATCCCGGCGCTGGATA
AAGAACTGAAAGCGAAAGGTAAGAGCGCGCTGATGTTCAACCTGCAAGAACCGTACTTCA
CCTGGCCGCTGATTGCTGCTGACGGGGGTTATGCGTTCAAGTATGAAAACGGCAAGTACG
ACATTAAAGACGTGGGCGTGGATAACGCTGGCGCGAAAGCGGGTCTGACCTTCCTGGTTG
ACCTGATTAAAAACAAACACATGAATGCAGACACCGATTACTCCATCGCAGAAGCTGCCTT
TAATAAAGGCGAAACAGCGATGACCATCAACGGCCCGTGGGCATGGTCCAACATCGACAC
CAGCAAAGTGAATTATGGTGTAACGGTACTGCCGACCTTCAAGGGTCAACCATCCAAACCG
TTCGTTGGCGTGCTGAGCGCAGGTATTAACGCCGCCAGTCCGAACAAAGAGCTGGCAAAA
GAGTTCCTCGAAAACTATCTGCTGACTGATGAAGGTCTGGAAGCGGTTAATAAAGACAAAC
CGCTGGGTGCCGTAGCGCTGAAGTCTTACGAGGAAGAGTTGGCGAAAGATCCACGTATTG
CCGCCACTATGGAAAACGCCCAGAAAGGTGAAATCATGCCGAACATCCCGCAGATGTCCG
CTTTCTGGTATGCCGTGCGTACTGCGGTGATCAACGCCGCCAGCGGTCGTCAGACTGTCG
ATGAAGCCCTGAAAGACGCGCAGACTAATGGGATCGAGGAAAACCTGTACTTCCAATCCAA
TGCACGCTTCCACGAACAATTTATTGTTCGCGAGGACCTGATGGGCTTGGCGATTGGCAC
CCACGGCGCGAACATTCAGCAGGCGCGTAAAGTCCCAGGTGTTACCGCTATTGATCTGGA
CGAAGACACGTGCACCTTTCATATTTATGGTGAAGACCAGGATGCTGTGAAAAAGGCGCGT
TCCTTCCTGGAATTTGCGGAAGACGTGATCCAGGTTCCGCGTAACCTGGTTGGTAAAGTCA
TCGGCAAAAACGGTAAGTTAATCCAAGAGATCGTGGACAAAAGCGGGGTAGTTCGCGTTC
GGATTGAAGCGGAAAATGAGAAGAACGTTCCGCAGGAGGAAGAAATTATGCCGCCAAATA
GCCTGCCGAGCAACAACTCACGTGTCGGTCCGAACGCTCCGGAAGAGAAGAAGCACCTG
GATATTAAAGAGAACAGCACCCATTTCAGCCAACCAAACTCCACTAAGGTGCAGCGTGTTT
TGGTAGCCAGCTCCGTTGTTGCCGGTGAGTCGCAAAAGCCGGAACTGAAAGCGTGGCAG
GGTATGGTGCCGTTCGTCTTTGTGGGCACCAAGGACAGCATCGCCAACGCAACGGTTCTG
CTGGACTACCATCTGAATTACCTGAAAGAAGTCGATCAGCTTCGTTTGGAACGCTTGCAAA
TCGATGAGCAACTGCGCCAGATCGGTGCGAGCTCTCGCCCGTCTCCGAACCGTACCGACA
AAGAGAAGAGCTACGTGACCGACGACGGTCAAGGTATGGGCCGTGGCAGCCGTCCGTAT
CGTAATAGGGGACATGGCCGCCGTGGTCCGGGTTACACCTCAGGTACGAACTCTGAGGC
GTCTAACGCCTCCGAAACCGAGTCGGATCATCGTGATGAGCTGAGCGACTGGTCACTGGC
GCCGACCGAAGAGGAGCGCGAGAGCTTTCTGGGTTCTTCTCACCATCACCATCACCATTA
A

MBP-(RGG+CTD)-His6

ATGGGTTCTTCTATGAAAATCGAAGAAGGTAAACTGGTAATCTGGATTAACGGCGATAAAG
GCTATAACGGTCTCGCTGAAGTCGGTAAGAAATTCGAGAAAGATACCGGAATTAAAGTCAC
CGTTGAGCATCCGGATAAACTGGAAGAGAAATTCCCACAGGTTGCGGCAACTGGCGATGG
CCCTGACATTATCTTCTGGGCACACGACCGCTTTGGTGGCTACGCTCAATCTGGCCTGTTG
GCTGAAATCACCCCGGACAAAGCGTTCCAGGACAAGCTGTATCCGTTTACCTGGGATGCC
GTACGTTACAACGGCAAGCTGATTGCTTACCCGATCGCTGTTGAAGCGTTATCGCTGATTT
ATAACAAAGATCTGCTGCCGAACCCGCCAAAAACCTGGGAAGAGATCCCGGCGCTGGATA
AAGAACTGAAAGCGAAAGGTAAGAGCGCGCTGATGTTCAACCTGCAAGAACCGTACTTCA
CCTGGCCGCTGATTGCTGCTGACGGGGGTTATGCGTTCAAGTATGAAAACGGCAAGTACG
ACATTAAAGACGTGGGCGTGGATAACGCTGGCGCGAAAGCGGGTCTGACCTTCCTGGTTG
ACCTGATTAAAAACAAACACATGAATGCAGACACCGATTACTCCATCGCAGAAGCTGCCTT
TAATAAAGGCGAAACAGCGATGACCATCAACGGCCCGTGGGCATGGTCCAACATCGACAC
CAGCAAAGTGAATTATGGTGTAACGGTACTGCCGACCTTCAAGGGTCAACCATCCAAACCG
TTCGTTGGCGTGCTGAGCGCAGGTATTAACGCCGCCAGTCCGAACAAAGAGCTGGCAAAA
GAGTTCCTCGAAAACTATCTGCTGACTGATGAAGGTCTGGAAGCGGTTAATAAAGACAAAC
CGCTGGGTGCCGTAGCGCTGAAGTCTTACGAGGAAGAGTTGGCGAAAGATCCACGTATTG
CCGCCACTATGGAAAACGCCCAGAAAGGTGAAATCATGCCGAACATCCCGCAGATGTCCG
CTTTCTGGTATGCCGTGCGTACTGCGGTGATCAACGCCGCCAGCGGTCGTCAGACTGTCG
ATGAAGCCCTGAAAGACGCGCAGACTAATGGGATCGAGGAAAACCTGTACTTCCAATCCAA
TGCACGTCGCGGTGATGGTCGCCGCAGAGGCGGTGGCGGTCGTGGCCAGGGCGGCCGT
GGCAGAGGCGGCGGTTTTAAAGGTAATGATGATCACAGCCGCACTGACAACCGTCCGCGT
AATCCGCGTGAGGCGAAGGGCAGAACTACAGATGGTAGCTTGCAAATCCGTGTGGACTGT
AATAACGAACGCAGCGTGCATACGAAAACCCTACAAAACACCAGTAGCGAGGGTAGCCGC
CTTCGTACCGGTAAAGACCGCAACCAGAAGAAAGAGAAACCGGACAGCGTTGATGGCCAA
CAACCGTTGGTGAATGGTGTTCCGGGTTCTTCTCACCATCACCATCACCATTAA

MBP-(RGG)-His6

ATGGGTTCTTCTATGAAAATCGAAGAAGGTAAACTGGTAATCTGGATTAACGGCGATAAAG
GCTATAACGGTCTCGCTGAAGTCGGTAAGAAATTCGAGAAAGATACCGGAATTAAAGTCAC
CGTTGAGCATCCGGATAAACTGGAAGAGAAATTCCCACAGGTTGCGGCAACTGGCGATGG
CCCTGACATTATCTTCTGGGCACACGACCGCTTTGGTGGCTACGCTCAATCTGGCCTGTTG
GCTGAAATCACCCCGGACAAAGCGTTCCAGGACAAGCTGTATCCGTTTACCTGGGATGCC
GTACGTTACAACGGCAAGCTGATTGCTTACCCGATCGCTGTTGAAGCGTTATCGCTGATTT
ATAACAAAGATCTGCTGCCGAACCCGCCAAAAACCTGGGAAGAGATCCCGGCGCTGGATA
AAGAACTGAAAGCGAAAGGTAAGAGCGCGCTGATGTTCAACCTGCAAGAACCGTACTTCA
CCTGGCCGCTGATTGCTGCTGACGGGGGTTATGCGTTCAAGTATGAAAACGGCAAGTACG
ACATTAAAGACGTGGGCGTGGATAACGCTGGCGCGAAAGCGGGTCTGACCTTCCTGGTTG
ACCTGATTAAAAACAAACACATGAATGCAGACACCGATTACTCCATCGCAGAAGCTGCCTT
TAATAAAGGCGAAACAGCGATGACCATCAACGGCCCGTGGGCATGGTCCAACATCGACAC
CAGCAAAGTGAATTATGGTGTAACGGTACTGCCGACCTTCAAGGGTCAACCATCCAAACCG
TTCGTTGGCGTGCTGAGCGCAGGTATTAACGCCGCCAGTCCGAACAAAGAGCTGGCAAAA
GAGTTCCTCGAAAACTATCTGCTGACTGATGAAGGTCTGGAAGCGGTTAATAAAGACAAAC
CGCTGGGTGCCGTAGCGCTGAAGTCTTACGAGGAAGAGTTGGCGAAAGATCCACGTATTG
CCGCCACTATGGAAAACGCCCAGAAAGGTGAAATCATGCCGAACATCCCGCAGATGTCCG
CTTTCTGGTATGCCGTGCGTACTGCGGTGATCAACGCCGCCAGCGGTCGTCAGACTGTCG
ATGAAGCCCTGAAAGACGCGCAGACTAATGGGATCGAGGAAAACCTGTACTTCCAATCCAA
TGCACGTCGCGGTGATGGTCGCCGCAGAGGCGGTGGCGGTCGTGGCCAGGGCGGCCGT
GGCAGAGGCGGCGGTTTTAAAGGTAATGATGATCACAGCCGCGGTTCTTCTCACCATCAC
CATCACCATTAA

MBP-(CTD)-His6
ATGGGTTCTTCTATGAAAATCGAAGAAGGTAAACTGGTAATCTGGATTAACGGCGATAAAG
GCTATAACGGTCTCGCTGAAGTCGGTAAGAAATTCGAGAAAGATACCGGAATTAAAGTCAC
CGTTGAGCATCCGGATAAACTGGAAGAGAAATTCCCACAGGTTGCGGCAACTGGCGATGG
CCCTGACATTATCTTCTGGGCACACGACCGCTTTGGTGGCTACGCTCAATCTGGCCTGTTG
GCTGAAATCACCCCGGACAAAGCGTTCCAGGACAAGCTGTATCCGTTTACCTGGGATGCC
GTACGTTACAACGGCAAGCTGATTGCTTACCCGATCGCTGTTGAAGCGTTATCGCTGATTT
ATAACAAAGATCTGCTGCCGAACCCGCCAAAAACCTGGGAAGAGATCCCGGCGCTGGATA
AAGAACTGAAAGCGAAAGGTAAGAGCGCGCTGATGTTCAACCTGCAAGAACCGTACTTCA
CCTGGCCGCTGATTGCTGCTGACGGGGGTTATGCGTTCAAGTATGAAAACGGCAAGTACG
ACATTAAAGACGTGGGCGTGGATAACGCTGGCGCGAAAGCGGGTCTGACCTTCCTGGTTG
ACCTGATTAAAAACAAACACATGAATGCAGACACCGATTACTCCATCGCAGAAGCTGCCTT
TAATAAAGGCGAAACAGCGATGACCATCAACGGCCCGTGGGCATGGTCCAACATCGACAC
CAGCAAAGTGAATTATGGTGTAACGGTACTGCCGACCTTCAAGGGTCAACCATCCAAACCG
TTCGTTGGCGTGCTGAGCGCAGGTATTAACGCCGCCAGTCCGAACAAAGAGCTGGCAAAA
GAGTTCCTCGAAAACTATCTGCTGACTGATGAAGGTCTGGAAGCGGTTAATAAAGACAAAC
CGCTGGGTGCCGTAGCGCTGAAGTCTTACGAGGAAGAGTTGGCGAAAGATCCACGTATTG
CCGCCACTATGGAAAACGCCCAGAAAGGTGAAATCATGCCGAACATCCCGCAGATGTCCG
CTTTCTGGTATGCCGTGCGTACTGCGGTGATCAACGCCGCCAGCGGTCGTCAGACTGTCG
ATGAAGCCCTGAAAGACGCGCAGACTAATGGGATCGAGGAAAACCTGTACTTCCAATCCAA
TGCAACTGACAACCGTCCGCGTAATCCGCGTGAGGCGAAGGGCAGAACTACAGATGGTAG
CTTGCAAATCCGTGTGGACTGTAATAACGAACGCAGCGTGCATACGAAAACCCTACAAAAC
ACCAGTAGCGAGGGTAGCCGCCTTCGTACCGGTAAAGACCGCAACCAGAAGAAAGAGAAA
CCGGACAGCGTTGATGGCCAACAACCGTTGGTGAATGGTGTTCCGGGTTCTTCTCACCAT
CACCATCACCATTAA
